# Tsc1-mTORC1 signaling controls striatal dopamine release and cognitive flexibility

**DOI:** 10.1038/s41467-019-13396-8

**Published:** 2019-11-28

**Authors:** Polina Kosillo, Natalie M. Doig, Kamran M. Ahmed, Alexander H.C.W. Agopyan-Miu, Corinna D. Wong, Lisa Conyers, Sarah Threlfell, Peter J. Magill, Helen S. Bateup

**Affiliations:** 1 Department of Molecular and Cell Biology, University of California, Berkeley, Berkeley, CA 94720 USA; 2 Medical Research Council Brain Network Dynamics Unit, University of Oxford, Oxford, OX1 3TH UK; 3 Department of Physiology, Anatomy and Genetics, University of Oxford, Oxford, OX1 3PT UK; 4 Oxford Parkinson’s Disease Centre, University of Oxford, Oxford, OX1 3QX UK; 5 Helen Wills Neuroscience Institute, University of California, Berkeley, Berkeley, CA 94720 USA; 6 Chan Zuckerberg Biohub, San Francisco, CA 94158 USA

**Keywords:** Cellular neuroscience, Developmental disorders, Neuronal physiology, Synaptic transmission

## Abstract

Tuberous Sclerosis Complex (TSC) is a neurodevelopmental disorder caused by mutations in *TSC1* or *TSC2*, which encode proteins that negatively regulate mTOR complex 1 (mTORC1). TSC is associated with significant cognitive, psychiatric, and behavioral problems, collectively termed TSC-Associated Neuropsychiatric Disorders (TAND), and the cell types responsible for these manifestations are largely unknown. Here we use cell type-specific *Tsc1* deletion to test whether dopamine neurons, which modulate cognitive, motivational, and affective behaviors, are involved in TAND. We show that loss of Tsc1 and constitutive activation of mTORC1 in dopamine neurons causes somatodendritic hypertrophy, reduces intrinsic excitability, alters axon terminal structure, and impairs striatal dopamine release. These perturbations lead to a selective deficit in cognitive flexibility, preventable by genetic reduction of the mTOR-binding protein Raptor. Our results establish a critical role for Tsc1-mTORC1 signaling in setting the functional properties of dopamine neurons, and indicate that dopaminergic dysfunction may contribute to cognitive inflexibility in TSC.

## Abstract (web summary)

Tuberous Sclerosis Complex (TSC) is a neurodevelopmental disorder caused by mutations in the *TSC1* or *TSC2* genes, which negatively regulate mTORC1 signalling. Here the authors selectively delete *Tsc1* from dopamine neurons in mice and find impairments in striatal dopamine release that are sufficient to reduce cognitive flexibility.

## Introduction

The mechanistic target of rapamycin (mTOR) is a ubiquitously expressed, conserved signaling cascade that integrates intra- and extracellular signals to regulate cellular metabolism and growth^1^. Deregulation of mTOR signaling is linked to numerous diseases, with particularly detrimental outcomes for nervous system development and function^2,3^. mTOR functions as a kinase within two complexes, mTORC1 and mTORC2, that have distinct protein components, upstream activators, and downstream targets^4^. Activation of mTORC1 promotes anabolic functions, including protein and lipid synthesis, and suppresses autophagy, leading to cell growth and division^1^. mTORC1 activity is negatively regulated by the heterodimeric Tsc1/2 protein complex that exerts GTP-ase-activating (GAP) activity on the small GTP-ase Rheb, a direct activator of mTORC1^5^. Disruption of the Tsc1/2 complex results in constitutively active mTORC1, leading to altered cell growth, metabolism, and proliferation^5^.

In post-mitotic neurons, Tsc1/2-mTORC1 signaling controls neuronal communication in a cell type-specific manner via regulation of membrane excitability, synaptic transmission, and synaptic plasticity^6–15^. Loss-of-function mutations in negative regulators of mTORC1 cause neurodevelopmental syndromes associated with significant neurological and psychiatric impairments. In particular, tuberous sclerosis complex (TSC), which results from mutations in *TSC1* or *TSC2*, causes early-onset epilepsy^16^. In addition, patients frequently present with neuropsychiatric conditions, including autism spectrum disorder (ASD), attention deficit hyperactivity disorder (ADHD), affective disorders, and cognitive deficits, collectively termed TAND (TSC-associated neuropsychiatric disorders)^17^. TAND is a major burden for patients^17^; however, little is known about how mutations in *TSC1/2* cause neuropsychiatric and behavioral abnormalities. In particular, the specific neuronal populations responsible for TAND and the functional consequences of mTOR pathway mutations on these neurons remain to be defined.

Midbrain dopamine (DA) neurons of the substantia nigra pars compacta (SNc) and ventral tegmental area (VTA) modulate a host of motivated behaviors^18–21^. Given the involvement of DA signaling in many of the neuropsychiatric conditions associated with TSC, we hypothesized that changes in DA signaling may contribute to TAND. To test this, we selectively deleted *Tsc1* from mouse DA neurons. This cell type-targeted approach allowed us to test whether constitutive activation of mTORC1 signaling in DA neurons alone was sufficient to drive TAND-related phenotypes.

Here we find that DA neurons are particularly sensitive to changes in mTORC1 signaling, as chronic activation of mTORC1 due to Tsc1 loss profoundly affects their structure and function resulting in significant impairments in striatal DA release. This dopaminergic deficit is associated with reduced cognitive flexibility in the absence of changes to motor or social behaviors. Our findings pinpoint mTORC1 as a critical regulator of midbrain DA neuron output and suggest that cognitive inflexibility in mTOR-related disorders may involve changes in DA signaling.

## Results

### Loss of Tsc1 causes DA neuron somatodendritic remodeling

**Fig. 1:**
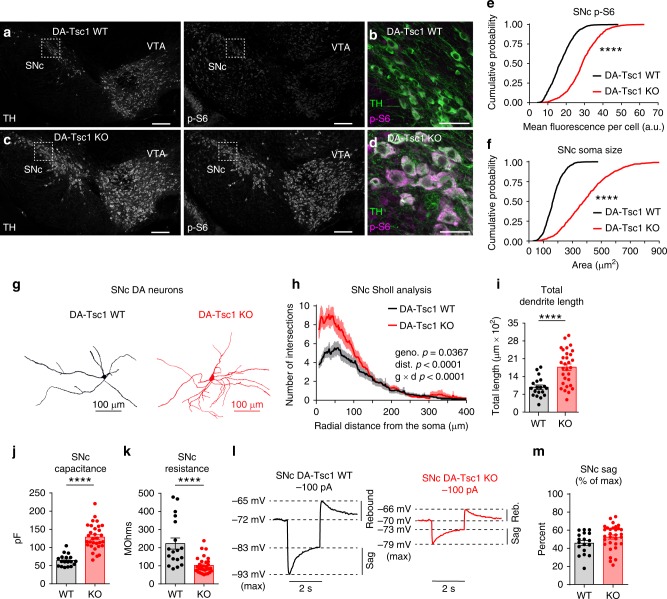
DA-Tsc1-KO SNc neurons are hypertrophic. **a**–**d** Confocal images of midbrain sections from DA-Tsc1-WT (**a**, **b**) and DA-Tsc1-KO (**c**, **d**) mice labeled with antibodies against tyrosine hydroxylase (TH) and phosphorylated S6 (p-S6, Ser240/244), scale bars = 200 μm. **b**, **d** show higher magnification merged images of the boxed regions in **a**, **c**, scale bars = 50 μm. **e**, **f** Cumulative probability plots of SNc DA neuron p-S6 levels (**e**) and soma area (**f**) (DA-Tsc1 WT: *n* = 759 neurons from three mice, DA-Tsc1 KO: *n* = 855 neurons from three mice). ****p < 0.0001, Kolmogorov–Smirnov tests. **g** Three-dimensional reconstructions of SNc DA neurons. **h** Sholl analysis of SNc DA neurons. Dark colored lines are the mean, lighter color shading represents SEM (DA-Tsc1 WT: *n* = 19 neurons from five mice, DA-Tsc1 KO: *n* = 30 neurons from nine mice). Two-way ANOVA *p* values are shown. **i** Mean ± SEM total dendritic length per cell (DA-Tsc1 WT: *n* = 19 neurons from five mice, DA-Tsc1 KO: *n* = 30 neurons from nine mice). ****p < 0.0001, Welch’s two-tailed *t* test. **j**, **k** Mean ± SEM membrane capacitance (**j**), and membrane resistance (**k**) (DA-Tsc1 WT: *n* = 18 neurons from five mice, DA-Tsc1 KO: *n* = 31 neurons from nine mice). ****p < 0.0001, Welch’s two-tailed *t* test (**j**) or Mann–Whitney test (**k**). **l** Example current-clamp recordings from SNc DA neurons in response to a −100 pA current step. **m** Mean ± SEM sag amplitude expressed as a percentage of the maximum hyperpolarization from baseline (DA-Tsc1 WT: *n* = 18 neurons from five mice, DA-Tsc1 KO: *n* = 31 neurons from nine mice). *p* = 0.0941, unpaired two-tailed *t* test. For all bar graphs, dots represent values for individual neurons. See also Supplementary Fig. 1 and Supplementary Tables 1 and 2. Source data are provided as a Source Data file.

The goal of this study was to test the sufficiency of manipulating mTORC1 signaling in DA neurons to drive cellular and behavioral phenotypes relevant to TAND. To do this, we conditionally deleted *Tsc1*^22^, which results in loss of function of the Tsc1/2 complex, from DA neurons using *DAT*^*IRES*^*Cre* mice^23^. Mice were heterozygous for *DAT*^*IRES*^*Cre* and had either wild-type (DA-Tsc1 WT) or homozygous floxed alleles of *Tsc1* (DA-Tsc1 KO) (Supplementary Fig. 1a, b). We quantified phosphorylation of the mTORC1 pathway target ribosomal protein S6 (p-S6) in tyrosine hydroxylase (TH)-labeled SNc and VTA neurons as a read-out of mTORC1 activity (Fig. 1a–e and Supplementary Fig. 1c, d). p-S6 levels were significantly higher in DA-Tsc1-KO neurons (Fig. 1e and Supplementary Fig. 1d), indicative of activated mTORC1 signaling. Consistent with the known function of mTORC1 in controlling cell size^1^, loss of Tsc1 caused a profound increase in DA neuron soma area (Fig. 1f and Supplementary Fig. 1e).

In addition to somatic hypertrophy, altered dendrite branching has been observed in neurons with mutations in mTORC1 regulators^9,14,24–27^. We performed Sholl analysis of neurobiotin-filled neurons (Supplementary Fig. 1f) and found that SNc and VTA DA neurons in DA-Tsc1-KO mice had more complex dendrites and increased total dendritic length (Fig. 1g–i and Supplementary Fig. 1g–i). Together, these data demonstrate that loss of Tsc1 strongly impacts the somatodendritic architecture of midbrain DA neurons.

### Tsc1 loss from DA neurons reduces intrinsic excitability

To test whether the extensive somatodendritic remodeling in DA-Tsc1-KO neurons resulted in a change to their membrane properties, we performed whole-cell recordings from DA neurons identified by a Cre-dependent tdTomato reporter^28^ (Supplementary Fig. 1b). Consistent with their hypertrophy, SNc and VTA DA-Tsc1-KO neurons had significantly increased membrane capacitance and decreased membrane resistance compared to WT neurons (Fig. 1j, k, Supplementary Fig. 1j, k, and Supplementary Tables 1 and 2). In response to hyperpolarization, midbrain DA neurons often exhibit a membrane potential sag mediated by hyperpolarization-activated cyclic nucleotide-gated (HCN) channels^29^. Although the absolute sag amplitude was significantly reduced in DA-Tsc1-KO neurons (Fig. 1l, Supplementary Fig. 1l), when expressed as a percentage of the maximum hyperpolarization from baseline, there was no difference between genotypes (Fig. 1m, Supplementary Fig. 1m and Supplementary Tables 1 and 2). This suggests that DA-Tsc1-KO neurons have normal HCN activation, but that changes in their passive membrane properties affect their overall response to hyperpolarizing current.

**Fig. 2:**
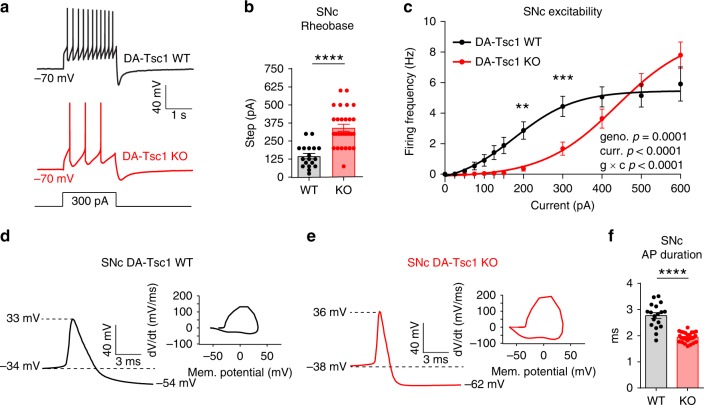
DA-Tsc1-KO SNc neurons are hypoexcitable. **a** Examples of action potential firing elicited with a 300 pA current step in SNc DA neurons. **b** Mean ± SEM rheobase of SNc DA neurons calculated as the current at which action potentials were first elicited (DA-Tsc1 WT: *n* = 18 neurons from five mice, DA-Tsc1 KO: *n* = 28 neurons from nine mice). ****p < 0.0001, Welch’s two-tailed *t* test. **c** Excitability curves showing the firing frequency of SNc DA neurons in response to current steps of increasing amplitude. Data are displayed as mean ± SEM (DA-Tsc1 WT: *n* = 20 neurons from five mice, DA-Tsc1 KO: *n* = 32 neurons from nine mice). Two-way ANOVA *p* values are shown. **p = 0.0014; ***p = 0.0002, Sidak’s multiple comparisons test. **d**, **e** Examples of individual action potentials evoked by positive current injection and their respective phase plots for SNc DA neurons in DA-Tsc1-WT (**d**) and DA-Tsc1-KO (**e**) mice. **f** Mean ± SEM action potential (AP) duration of SNc DA neurons (DA-Tsc1 WT: *n* = 18 neurons from five mice, DA-Tsc1-KO: *n* = 28 neurons from nine mice). ****p < 0.0001, Welch’s two-tailed *t* test. For all bar graphs, dots represent values for individual neurons. See also Supplementary Fig. 1 and Supplementary Tables 1 and 2. Source data are provided as a Source Data file.

To measure intrinsic excitability, we injected steps of depolarizing current to generate excitability curves and found that SNc and VTA DA-Tsc1-KO cells were hypoexcitable (Fig. 2a–c, Supplementary Fig. 1n–p, and Supplementary Tables 1 and 2). Significantly more current was required to evoke action potential (AP) firing in DA-Tsc1-KO neurons (Fig. 2b and Supplementary Fig. 1o). At current step amplitudes up to 400 pA, DA-Tsc1-KO neurons fired fewer APs than DA-Tsc1-WT neurons; however, at ≥500 pA current, DA-Tsc1-KO neurons had similar or increased firing (Fig. 2c and Supplementary Fig. 1p). Thus, more current is required to drive spiking in DA-Tsc1-KO neurons, but these neurons may be able to fire a burst of APs given a sufficiently large excitatory input.

DA neurons have been classically defined by their relatively long AP duration and prominent after-hyperpolarization^30^. We examined the properties of single APs evoked by positive current injection and found that *Tsc1* deletion substantially changed the AP waveform (Fig. 2d, e, Supplementary Fig. 1q, r and Supplementary Tables 1 and 2). Specifically, the AP duration of SNc and VTA DA-Tsc1-KO neurons was significantly shorter (Fig. 2f and Supplementary Fig. 1s), which may be due to altered potassium channels that control repolarization^31^. These electrophysiology changes are similar to those of *Tsc1*-KO thalamic neurons, which also showed altered passive membrane properties and shorter duration APs^11^.

### Tsc1 loss impairs striatal DA release

**Fig. 3:**
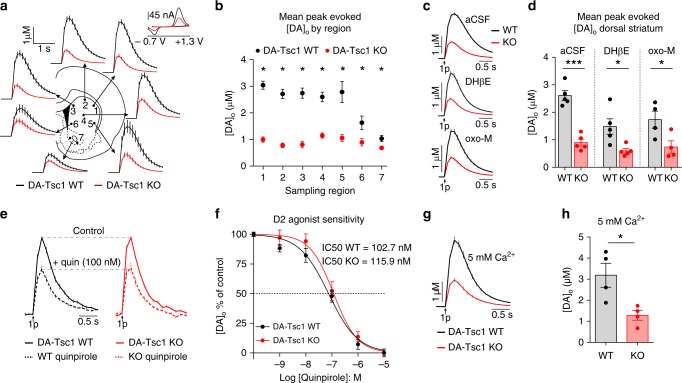
Loss of Tsc1 impairs striatal DA release. **a** Mean ± SEM [DA]_o_ versus time evoked from different striatal subregions by a single electrical pulse. Traces are an average of 16–36 transients per site from five mice per genotype. Inset, typical cyclic voltammograms show characteristic DA waveform. **b** Mean ± SEM peak [DA]_o_ by striatal region (numbers correspond to the sites in **a**). *n* = 16–36 transients per site from five mice per genotype. *p_1–4_ <0.0001; *p_6_ = 0.0067, paired two-tailed *t* tests; *p_5_ = 0.0002; *p_7_ = 0.0175, Wilcoxon’s two-tailed *t* tests. **c** Mean ± SEM [DA]_o_ versus time averaged across all dorsal striatum sites (sites #1–6) recorded in aCSF (average of 100 transients across six recording sites from five mice per genotype), 1 μM DHβE (average of 118–122 transients across six recording sites from five mice per genotype), or 10 μM oxotremorine-M (oxo-M, average of 86–88 transients across six recording sites from four mice per genotype). **d** Mean ± SEM peak [DA]_o_ averaged across all dorsal striatum sites (sites #1–6) recorded in aCSF (*n* = 5 mice per genotype), 1 μM DHβE (*n* = 5 mice per genotype) or 10 μM oxotremorine-M (*n* = 4 mice per genotype). ***p_aCSF_ = 0.0002; *p_DHβE_ = 0.0126; *p_oxo-M_ = 0.0479, paired two-tailed *t* tests. **e** Mean ± SEM [DA]_o_ versus time in the dorsal striatum (site #4) in control conditions (solid lines) and in 100 nM quinpirole (dashed lines). Average of 18 transients per drug concentration from three mice per genotype. **f** Mean ± SEM quinpirole dose–response curves, sigmoidal curve fit. *n* = 3 mice per genotype. **g** Mean ± SEM [DA]_o_ versus time across all dorsal striatum sites (sites #1–6) in 5 mM Ca^2+^. Traces are an average of 96 transients across six recording sites from four mice per genotype. **h** Mean ± SEM peak [DA]_o_ in 5 mM Ca^2+^ averaged across all dorsal striatum sites (sites #1–6). *n* = 4 mice per genotype. *p = 0.0128, paired two-tailed *t* test. For all bar graphs, dots represent values for individual mice. For **e**–**h**, 1 μM DHβE was applied. See also Supplementary Fig. 2. Source data are provided as a Source Data file.

The AP firing of DA neurons does not linearly translate into axonal DA release^32,33^. Therefore, to test how loss of Tsc1 affects DA output, we used fast-scan cyclic voltammetry (FCV) to monitor electrically evoked DA release in the striatum. We found a significant decrease in the peak amplitude of evoked extracellular DA ([DA]_o_) released throughout the dorsal striatum and, to a lesser extent, in the nucleus accumbens (NAc) core, in DA-Tsc1-KO mice (Fig. 3a, b). Decreased DA release was found with both single-pulse (Fig. 3a, b) and high-frequency burst stimulation (four pulses at 100 Hz, Supplementary Fig. 2a, b). Combining all dorsal striatal sites, we found an average 60% reduction in single pulse (Fig. 3c, d) and 45% reduction in high-frequency burst-evoked [DA]_o_ (Supplementary Fig. 2c, d). We tested multiple electrical stimulation intensities and found that DA release deficits in DA-Tsc1-KO slices were exacerbated at low currents, consistent with their intrinsic hypoexcitability, while peak-evoked [DA]_o_ plateaued at similar stimulation intensities as in WT slices (Supplementary Fig. 2e). Notably, the dorsal striatal release deficits in DA-Tsc1-KO mice did not deteriorate further in aged animals and remained ~60% below WT levels in 22-month-old mice (Supplementary Fig. 2f–h).

Striatal DA release is tightly regulated by striatal cholinergic interneurons (ChIs)^32,33^. Therefore, we repeated the FCV experiments with cholinergic transmission blockers to test for potential involvement of ChIs in the release phenotypes (Supplementary Fig. 2i). We found that release deficits in DA-Tsc1-KO slices generally persisted after blockade of cholinergic transmission (Fig. 3c, d and Supplementary Fig. 2c, d), confirming a cell autonomous effect on DA neuron function.

### Autoreceptor occupancy and calcium sensitivity are unaltered

One possible mechanism for a reduction in striatal [DA]_o_ is increased activity or expression of type 2 DA (D2) autoreceptors on DA axons, which inhibit DA release^33,34^ (Supplementary Fig. 2j). Application of the D2 receptor agonist quinpirole decreased peak-evoked [DA]_o_ to a similar extent in DA-Tsc1-WT and DA-Tsc1-KO slices (WT half-maximal inhibitory concentration (IC_50_) = 102.7 ± 0.79 nM, KO IC_50_ = 115.9 ± 0.81 nM, Fig. 3e, f). Therefore, it is unlikely that changes in D2 autoreceptor function account for the impairment in evoked DA release.

Striatal DA release is dependent on voltage-gated calcium channels and is thus sensitive to changes in extracellular calcium levels^32,33^. We tested if increasing extracellular calcium could ameliorate release deficits. As expected, increasing extracellular calcium to 5 mM led to increased peak-evoked [DA]_o_ in both genotypes compared to normal 2.4 mM Ca^2+^. However, dorsal striatal DA release in DA-Tsc1-KO slices remained ~60% lower than in WT slices (Fig. 3g, h). We conclude that altered calcium sensitivity is unlikely to be a primary contributor to the release deficits observed.

### DA release probability is decreased following *Tsc1* deletion

**Fig. 4:**
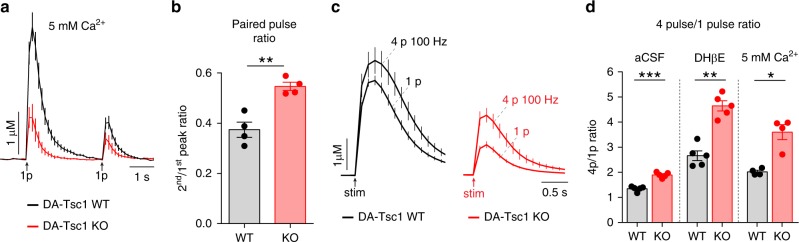
Loss of Tsc1 reduces striatal DA release probability. **a** Mean ± SEM dorsolateral striatal [DA]_o_ versus time evoked by single-pulse stimulations (1*p*) delivered 3 s apart in 5 mM extracellular Ca^2+^ (1 μM DHβE applied throughout). Traces are an average of 16 transients per genotype from four mice. **b** Mean ± SEM ratio of peak [DA]_o_ in the dorsolateral striatum evoked by the second-to-first pulse in 5 mM extracellular Ca^2+^ (1 μM DHβE applied throughout). **p = 0.0055, paired two-tailed *t* test. **c** Mean ± SEM [DA]_o_ versus time evoked by single-pulse (1*p*) or high-frequency stimulation (four pulses at 100 Hz, 4*p*) in the dorsolateral striatum in aCSF. Traces are an average of 18 transients for 1*p* and nine transients for 4*p* from five mice per genotype. **d** Mean ± SEM ratio of peak [DA]_o_ evoked by a four-pulse train to single-pulse stimulation in the dorsolateral striatum in aCSF (*n* = 5 mice per genotype), 1 μM DHβE (*n* = 5 mice per genotype), or 5 mM extracellular Ca^2+^ (*n* = 4 mice per genotype). *p = 0.0204; **p = 0.0024; ***p = 0.0009, paired two-tailed *t* tests. For all bar graphs, dots represent values for individual mice. See also Supplementary Fig. 2. Source data are provided as a Source Data file.

The amount of DA released upon stimulation is dependent upon release probability (*P*_r_)^35,36^. While DA neurons have distinct release machinery and properties compared to fast-acting neurotransmitters^37^, an estimate of DA *P*_r_ can be generated by examining the responses to multiple stimuli^36^. We measured the paired-pulse ratio of dorsal striatal release events evoked by single stimuli delivered 3 s apart, in the presence of dihydro-β-erythroidine (DHβE) to remove cholinergic modulation and 5 mM Ca^2+^ to maximize *P*_r_ (Fig. 4a). The second-to-first peak ratio was significantly higher in DA-Tsc1-KO slices (Fig. 4b), indicative of a reduction in initial *P*_r_. We also compared the ratio of peak [DA]_o_ evoked by four stimuli delivered at 100 Hz to that evoked by a single pulse (4*p*/1*p* ratio) in the dorsal striatum (Fig. 4c). The 4*p*/1*p* ratio was significantly higher in DA-Tsc1-KO slices compared to WT across experimental conditions (Fig. 4d). These results are again consistent with reduced initial *P*_r_, a conclusion supported by the increased frequency-response sensitivity observed in DA-Tsc1-KO slices (Supplementary Fig. 2k).

In the course of the FCV experiments, we observed that the clearance of [DA]_o_, which is mediated by re-uptake of DA back into the axon terminal by the DA active transporter (DAT), was faster in DA-Tsc1-KO slices (Supplementary Fig. 2l, m). This enhanced re-uptake, combined with the initial release deficit due to reduced *P*_r_, indicates that loss of Tsc1 from DA neurons strongly impairs their ability to influence dorsal striatal targets.

### DA production is increased following Tsc1 loss

**Fig. 5:**
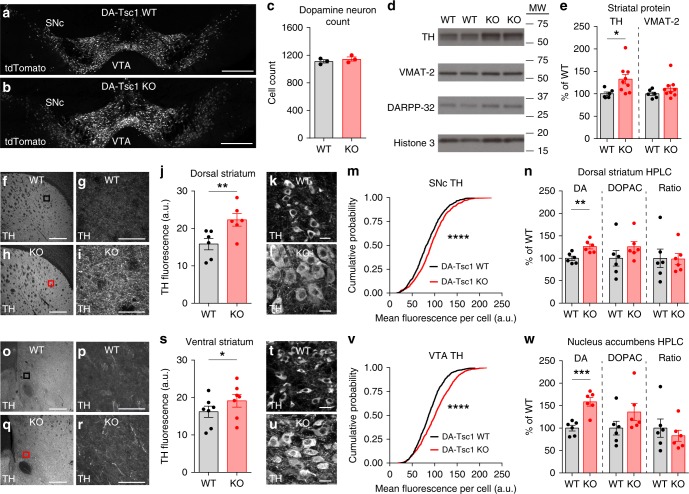
DA-Tsc1-KO mice have increased DA synthesis. **a**, **b** Confocal images of midbrain sections from DA-Tsc1-WT (**a**) and DA-Tsc1-KO (**b**) mice expressing a Cre-dependent tdTomato reporter in DA neurons, scale bars = 500 μm. **c** Mean ± SEM number of tdTomato-positive DA neurons, SNc and VTA combined. *n* = 3 mice per genotype, *p* = 0.4343, unpaired, two-tailed *t* test. **d** Representative Western blots for tyrosine hydroxylase (TH), vesicular monoamine transporter (VMAT-2), DARPP-32, and histone 3. **e** Mean ± SEM striatal protein content of TH and VMAT-2. *n* = 6 DA-Tsc1-WT and 9 DA-Tsc1-KO mice. *p = 0.0169, Welch’s two-tailed *t* test. **f**–**i** Low-magnification image (**f**, **h**, scale bars = 400 μm) and high-magnification image (**g**, **i**, from the boxed regions, scale bars = 50 μm) of the dorsolateral striatum from DA-Tsc1-WT (**f**, **g**) and DA-Tsc1-KO (**h**, **i**) mice showing TH immunostaining. **j** Mean ± SEM TH immunofluorescence intensity in the dorsolateral striatum. *n* = 6 mice per genotype. **p = 0.0055, paired two-tailed *t* test. **k**, **l** Images of SNc DA neurons from DA-Tsc1-WT (**k**) or DA-Tsc1-KO (**l**) mice immunostained for TH, scale bars = 25 μm. **m** Cumulative distributions of TH levels in SNc DA neurons (DA-Tsc1 WT: *n* = 759 neurons from three mice, DA-Tsc1 KO: *n* = 855 neurons from three mice). ****p < 0.0001, Kolmogorov–Smirnov test. **n** Dorsal striatum mean ± SEM total tissue content of DA and 3,4-dihydroxyphenylacetic acid (DOPAC). Ratio = DOPAC/DA per mouse. *n* = 6 mice per genotype, **p = 0.0043, unpaired, two-tailed *t* test. **o**–**r** Low-magnification image (**o**, **q**, scale bars = 400 μm) and high-magnification image (**p**, **r**, from the boxed regions, scale bars = 50 μm) of the ventral striatum from DA-Tsc1-WT (**o**, **p**) and DA-Tsc1-KO (**q**, **r**) mice showing TH immunostaining. **s** Mean ± SEM TH immunofluorescence intensity in the ventral striatum. *n* = 7 mice per genotype. *p = 0.0159, paired, two-tailed *t* test. **t**, **u** Confocal images of VTA DA neurons from DA-Tsc1-WT (**t**) or DA-Tsc1-KO (**u**) mice immunostained for TH, scale bars = 25 μm. **v** Cumulative distributions of TH levels in VTA DA neurons (DA-Tsc1 WT: *n* = 892 neurons from three mice, DA-Tsc1 KO: *n* = 863 neurons from three mice). ****p < 0.0001, Kolmogorov–Smirnov test. **w** Nucleus accumbens mean ± SEM total tissue content of DA and DOPAC. *n* = 6 mice per genotype. ***p = 0.0004, unpaired, two-tailed *t* test. For all bar graphs, dots represent values for individual mice. Source data are provided as a Source Data file.

We next investigated whether the reduction in evoked DA release was a result of decreased numbers of DA neurons, reduced striatal innervation, and/or impaired DA production. At 3 months of age, the number of DA neurons in the midbrain was not significantly different between littermate DA-Tsc1-WT and DA-Tsc1-KO mice (Fig. 5a–c, *p* = 0.4343, unpaired, two-tailed *t* test). Therefore, deletion of *Tsc1* around embryonic day 17^23^ does not strongly impact the differentiation or survival of DA neurons into adulthood. We next examined whether striatal axons in DA-Tsc1-KO mice had the capacity to synthesize and package DA. We found that dopaminergic innervation of both the dorsal and ventral striatum was intact and expression of the vesicular monoamine transporter-2 (VMAT-2), responsible for packaging DA into vesicles, was normal in total striatal homogenates (Fig. 5d, e). Interestingly, we found that DA-Tsc1-KO striatal axons and DA cell bodies in both the SNc and VTA had higher expression of TH, the rate-limiting enzyme in DA synthesis (Fig. 5d–m, o–v). We tested whether higher TH levels resulted in increased DA synthesis by analyzing total tissue DA content using high-performance liquid chromatography (HPLC). The total content of DA was significantly elevated in DA-Tsc1-KO mice in both dorsal and ventral striatal regions, while levels of the primary DA metabolite 3,4-dihydroxyphenylacetic acid (DOPAC) were not significantly affected (Fig. 5n, w). Since the ratio of DOPAC to DA per mouse was unchanged by loss of Tsc1 (Fig. 5n, w), we conclude that the high DA levels were due to increased DA synthesis rather than reduced turnover.

### Loss of Tsc1 alters DA axon ultrastructure

**Fig. 6:**
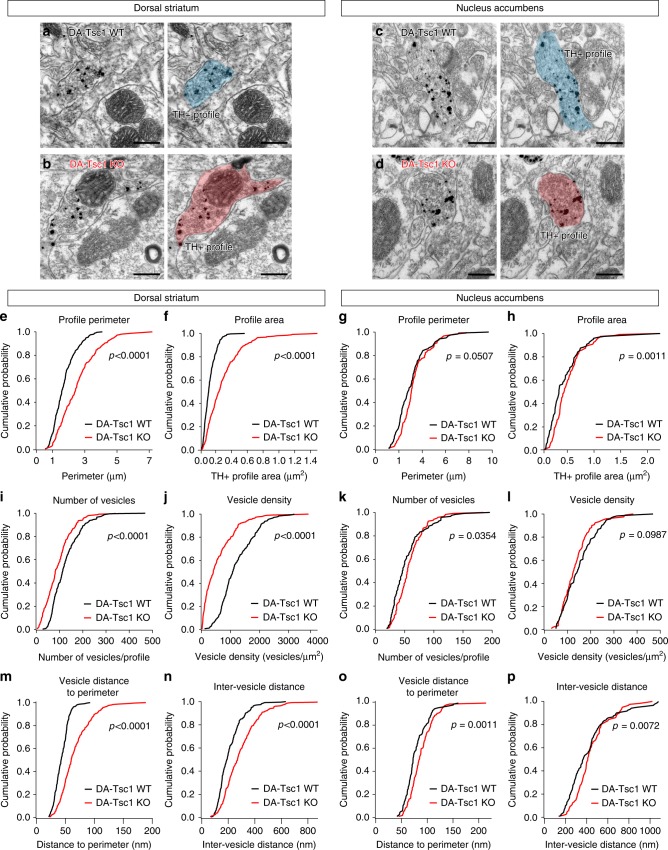
Loss of Tsc1 alters DA axon ultrastructure. **a**–**d** Example electron micrographs of dopaminergic axon profiles in the dorsolateral striatum (**a**, **b**) or nucleus accumbens core (**c**, **d**) of DA-Tsc1-WT (**a**, **c**) and DA-Tsc1-KO (**b**, **d**) mice, identified by enriched immunogold labeling for tyrosine hydroxylase (TH), scale bars = 0.2 μm. Right panels show TH + axon profiles pseudocolored in blue (DA-Tsc1 WT) or red (DA-Tsc1 KO). **e**–**p** Cumulative distributions of TH + axon profile perimeter (**e**, **g**), profile area (**f**, **h**), number of vesicles per profile (**i**, **k**), vesicle density (**j**, **l**), vesicle distance to plasma membrane (**m**, **o**), and inter-vesicle distance (**n**, **p**). *p* Values for Kolmogorov–Smirnov tests are shown. For **e**, **f**, **i**, **j**, **m**, **n**: DA-Tsc1 WT, *n* = 239 terminals (synaptic and non-synaptic) from four mice; DA-Tsc1 KO, *n* = 252 terminals from four mice. For **g**, **h**, **k**, **l**, **o**, **p**: *n* = 120 terminals (synaptic and non-synaptic) from four mice per genotype. Source data are provided as a Source Data file.

Our analyses revealed a strong impairment in dorsal but not ventral striatal DA release (see Fig. 3) despite elevated DA tissue content in both regions (see Fig. 5). This prompted us to investigate whether striatal subregion-specific structural changes could be responsible for the DA release deficits. We performed electron microscopy (EM) analysis of DA axons identified by TH immunogold labeling in the dorsolateral striatum or NAc core. DA neuron axons arborize extensively in the striatum, releasing DA at both synaptic and non-synaptic sites^38–40^. We found that the majority of TH + axon profiles were non-synaptic, defined by the lack of a discernable synaptic membrane specialization (Fig. 6a–d). The proportion of axon profiles that formed synapses was similar between genotypes (Supplementary Fig. 3a–e and Supplementary Tables 3 and 4). Both synaptic and non-synaptic axon profiles were included in the main analysis. Supplementary Tables 3 and 4 provide full EM results including separate analyses of axon profiles forming synapses.

Consistent with the somatodendritic hypertrophy of SNc neurons (see Fig. 1), TH + axonal profile perimeter and area were significantly increased in the dorsal striatum of DA-Tsc1-KO mice (Fig. 6e, f). However, the TH + axon profiles in the NAc, which mainly arise from VTA neurons^41^, were not strongly impacted (Fig. 6g, h). We quantified the number of vesicles associated with each axon profile and found that vesicle number was significantly decreased in DA-Tsc1-KO profiles in the dorsal striatum, leading to a large reduction in vesicle density (Fig. 6i, j). By contrast, vesicle number in axon profiles in the NAc was slightly increased in DA-Tsc1-KO mice (Fig. 6k), and there was no significant change in vesicle density between genotypes (Fig. 6l).

To determine the spatial arrangement of vesicles within the axonal profile, we measured the shortest distance of each vesicle to the plasma membrane, and the distance of each vesicle to every other vesicle within the profile (Supplementary Fig. 3f). The average vesicle distance to the plasma membrane and inter-vesicle distance within each profile were significantly increased in DA-Tsc1-KO mice in both the dorsal striatum and NAc, but the effects were more pronounced in the dorsal striatum (Fig. 6m–p). When plotted as a histogram, the distribution of inter-vesicle distances was shifted to the right for DA-Tsc1-KO profiles, with a significant interaction between genotype and inter-vesicle distance found only for dorsal striatal terminals (Supplementary Fig. 3g, h).

Taken together, the EM analyses revealed profound axon profile hypertrophy and reduced vesicle density and clustering in the dorsal striatum of DA-Tsc1-KO mice. Since the TH + axon profiles in the NAc from the same DA-Tsc1-KO mice were either not significantly changed or only modestly affected, it is likely that these selective changes in axonal architecture underpin the observed striatal region-specific deficits in DA release (see Fig. 3).

### Motor and social behaviors are unaffected in DA-Tsc1-KO mice

DA is involved in a variety of behaviors, from movement to reward learning to cognition^18–21^. To determine how the cellular changes described above impact behavior in DA-Tsc1-KO mice, we performed assays that are sensitive to dopaminergic function and relevant to TAND. Supplementary Table 5 reports all behavior results and Supplementary Table 6 shows behavioral analysis by sex.

To examine locomotor activity, we tested animals in the open field (Supplementary Fig. 4a). Deletion of *Tsc1* did not affect exploratory locomotor behavior, measured as the distance traveled in the first 10 min (DA-Tsc1 WT: 24.55 ± 1.84 m, DA-Tsc1 KO: 23.02 ± 1.49 m, *p* = 0.5153, unpaired, two-tailed *t* test). The total distance traveled over 60 min and the mean speed were also similar between genotypes (Supplementary Fig. 4b, c). We observed no changes in rearing behavior or time spent grooming (Supplementary Fig. 4d, e). Additionally, home cage locomotor activity (Supplementary Fig. 4h–k) and spontaneous stereotypies were not strongly impacted by Tsc1 loss (Supplementary Tables 5 and 6).

To test whether motor coordination or motor learning were altered in DA-Tsc1-KO mice, we performed the accelerating rotarod test^42^ (Supplementary Fig. 4l, m). We found no significant differences in either initial motor coordination (DA-Tsc1 WT 10.55 ± 1.19 r.p.m., DA-Tsc1 KO 13.37 ± 1.39 r.p.m., *p* = 0.2904, Mann–Whitney test, both sexes combined) or motor learning (DA-Tsc1 WT 1.92 ± 0.25 r.p.m. per day, DA-Tsc1 KO 1.32 ± 0.19 r.p.m. per day, *p* = 0.0637, unpaired *t* test, both sexes combined). Together, these results indicate that general locomotor behaviors were unaffected by DA neuron-specific loss of *Tsc1*.

DA signaling modulates affective behaviors including anxiety and sociability^43,44^, which can be impacted in patients with TSC^17,45^. To measure avoidance behavior, we tested mice in the elevated plus maze and found no significant difference in the number of entries into or time spent in the open or closed arms (Supplementary Fig. 4n–q). We did, however, find a small but significant decrease in the time spent in the center of the open field in male DA-Tsc1-KO mice, which indicates greater avoidance behavior (Supplementary Fig. 4g). Social approach was normal in DA-Tsc1-KO mice as assessed by the three-chamber test (Supplementary Fig. 4r, s). Since these behaviors are largely mediated by mesolimbic DA circuits arising from the VTA^43,44^, the lack of major changes may reflect the relative sparing of NAc DA release in DA-Tsc1-KO mice (see Fig. 3).

### DA-Tsc1-KO mice exhibit reduced cognitive flexibility

**Fig. 7:**
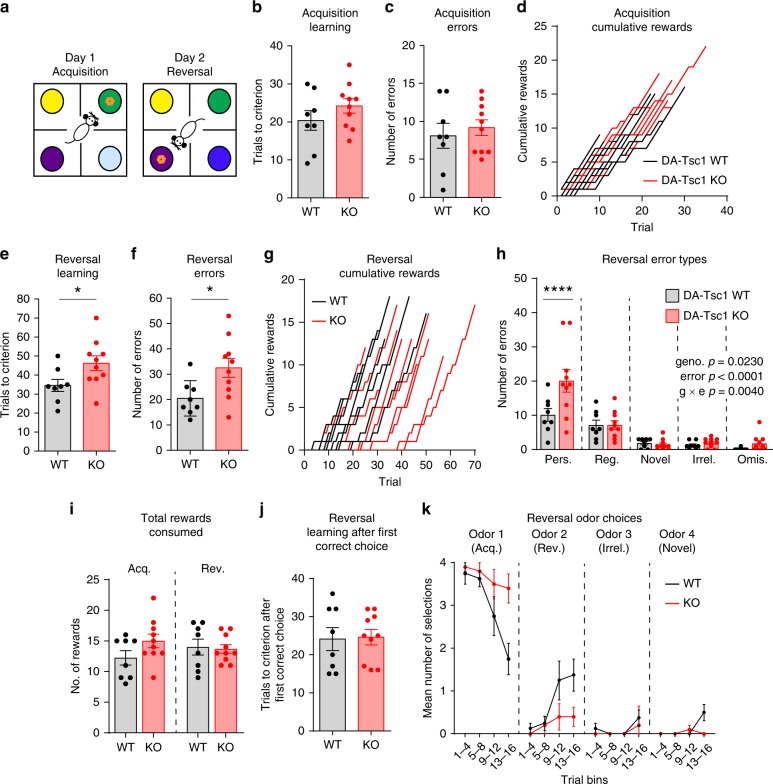
DA-Tsc1-KO mice exhibit reduced cognitive flexibility. **a** Schematic of the four-choice odor-based reversal learning task. Orange ring denotes the food reward. Dark blue circle in Reversal indicates a novel odor. **b**, **c** Mean ± SEM number of trials taken to reach criterion (eight out of ten correct) during acquisition learning (**b**) and total errors made during acquisition learning (**c**) in the four-choice test. **d** Step function cumulative reward plot for individual animals during acquisition learning. **e**, **f** Mean ± SEM number of trials taken to reach criterion (eight out of ten correct) during reversal learning (**e**, *p = 0.0392) and total errors made during reversal learning (**f**, *p = 0.0230), unpaired, two-tailed *t* tests. **g** Step function cumulative reward plot for individual animals during reversal learning. **h** Analysis of different error types during reversal learning. Bars represent mean ± SEM number of perseverative (Pers.), regressive (Reg.), novel, irrelevant (Irrel.), or omission (Omis.) errors for mice of each genotype. Two-way ANOVA *p* values are shown. ****p < 0.0001, Sidak’s multiple comparisons test. **i** Mean ± SEM total number of rewards obtained and consumed during acquisition (Acq.) and reversal (Rev.) learning. **j** Mean ± SEM and number of trials to reach criterion (eight out of ten correct) during reversal learning after the first correct selection of the newly rewarded odor. **k** Mean ± SEM number of odor selections in bins of 4 trials during reversal learning. Odor 1 is the odor rewarded during acquisition learning and odor 2 is the odor rewarded during reversal learning. For all panels DA-Tsc1-WT: *n* = 8 male mice and DA-Tsc1-KO: *n* = 10 male mice. For all bar graphs, dots represent values for individual mice. See Supplementary Table 5 for *p* values for all behavior tests. Source data are provided as a Source Data file.

Cognitive flexibility is commonly disrupted in TSC^17^ and in several neuropsychiatric conditions including ASD and ADHD^18,46,47^. Dorsal striatal DA signaling has been implicated in cognitive flexibility^48,49^, and decreased striatal DA is associated with reduced cognitive performance^50–52^. We therefore hypothesized that loss of Tsc1 from DA neurons may disrupt cognitive flexibility. To examine associative learning and cognitive flexibility, we used the four-choice odor-based reversal task (Fig. 7a)^53^, in which reversal learning is sensitive to changes in DA neuron function^54^. We found that male DA-Tsc1-KO mice exhibited intact acquisition learning, reaching criterion in a similar number of trials (Fig. 7b) and making, on average, the same number of errors as littermate controls (Fig. 7c). Plots of the cumulative rewards received by individual mice across trials showed that the genotypes were overlapping (Fig. 7d). By contrast, when the rewarded odor was changed the following day, DA-Tsc1-KO mice took, on average, significantly longer to reach criterion and made more total errors (Fig. 7e, f). The time course of cumulative rewards during reversal learning shows that half of the DA-Tsc1-KO mice went more than 20 trials before earning a reward, while all DA-Tsc1-WT mice had received a reward by trial 20 (Fig. 7g). For both genotypes, the majority of errors were perseverative (Fig. 7h), meaning that the mice continued to choose the initially learned odor even when it was no longer rewarded. However, DA-Tsc1-KO mice made significantly more perseverative errors than WT littermates (Fig. 7h). Thus, DA-Tsc1-KO mice exhibited a selective deficit in cognitive flexibility characterized by perseveration, wherein they were slower to update their behavioral strategy in the face of changed environmental demands.

It is unlikely that these findings were due to alterations in odor preference or sensitivity as mice of both genotypes showed similar spontaneous odor preference in the first four trials of acquisition learning and could clearly discriminate between the four odors prior to making a digging choice. Differences in hunger status or motivation were also unlikely as both groups consumed a similar number of total rewards during acquisition and reversal (Fig. 7i). Additionally, during reversal learning, once the DA-Tsc1-KO mice made their first correct odor choice, they took a similar number of trials to reach criterion as DA-Tsc1-WT mice (Fig. 7j). These data indicate that perseveration to choose the initially rewarded odor at the expense of exploring other odors was the main driver for the slower reversal learning in DA-Tsc1-KO mice. This is further supported by plotting the average odor choices in four trial bins during the first 16 trials of reversal learning (Fig. 7k). This analysis shows that DA-Tsc1-KO mice continue to choose the initially rewarded odor for longer, while DA-Tsc1-WT mice are faster to switch their odor choice. Together, these results demonstrate that loss of Tsc1 from DA neurons is sufficient to increase perseveration and reduce cognitive flexibility in mice.

### Systemic rapamycin does not suppress mTORC1 in DA neurons

To test whether phenotypes observed in DA-Tsc1-KO mice could be rescued by pharmacologic inhibition of mTOR signaling, we treated mice chronically with rapamycin (8 mg/kg per day, 3 days a week) for 2 months starting at 4 weeks of age. We quantified p-S6 levels as a read-out of mTORC1 signaling, and found, surprisingly, that rapamycin treatment did not reduce p-S6 levels in DA neurons compared to vehicle in either DA-Tsc1-WT or DA-Tsc1-KO mice (Supplementary Fig. 5). Importantly, however, when we analyzed hippocampal and cerebellar neurons from the same mice, we found the expected decrease in p-S6 levels after rapamycin treatment (Supplementary Fig. 6), consistent with prior studies^7,13^. This demonstrates that systemic rapamycin has differential effects depending on the cell type and suggests that chronic rapamycin may not be an effective treatment to decrease mTORC1 signaling and ameliorate phenotypic changes in DA neurons after Tsc1 loss.

### Genetic reduction of *Rptor* constrains mTORC1 signaling

**Fig. 8:**
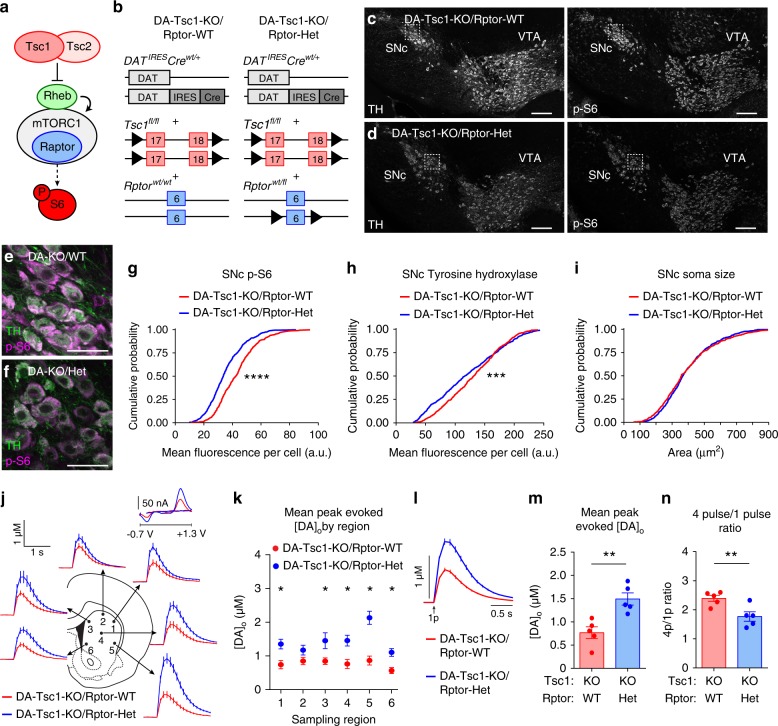
Heterozygous *Rptor* deletion prevents DA release deficits in DA-Tsc1-KO mice. **a** Simplified mTORC1 signaling schematic showing the Tsc1/2 heterodimer as a negative regulator of the small GTP-ase Rheb, which directly promotes mTORC1 kinase activity. Raptor is an essential component of mTORC1. **b** Schematic of the genetic strategy to selectively delete *Tsc1* and reduce *Rptor* by one copy in DA neurons. **c**–**f** Confocal images of midbrain sections from DA-Tsc1-KO/Rptor-WT (**c**, **e**) and DA-Tsc1-KO/Rptor-Het (**d**, **f**) mice immunostained for tyrosine hydroxylase (TH) and phosphorylated S6 (p-S6, ser240/244), scale bars = 200 μm. **e**, **f** show higher magnification merged images of the boxed regions in **c**, **d**, scale bars = 50 μm. **g**–**i** Cumulative distributions of SNc DA neuron p-S6 levels (**g**), tyrosine hydroxylase levels (**h**), and soma area (**i**) (DA-Tsc1-KO/Rptor-WT: *n* = 509 neurons from three mice, DA-Tsc1-KO/Rptor-Het: *n* = 567 neurons from three mice). ****p < 0.0001; ***p = 0.0003, Kolmogorov–Smirnov tests. **j** Mean ± SEM [DA]_o_ versus time evoked by a single electrical pulse from different dorsal striatal subregions. Traces show an average of 17–20 transients per recording site from 5 mice per genotype. Inset, typical cyclic voltammograms show characteristic DA waveform. **k** Mean ± SEM peak [DA]_o_ by striatal region (numbers correspond to the sites in **j**). *n* = 17–20 transients per recording site from five mice per genotype. *p_1_ = 0.0002; *p_3_ = 0.0.0150; *p_4_ = 0.0033; *p_5_ < 0.0001; *p_6_ = 0.0005, paired two-tailed *t* tests. **l** Mean ± SEM [DA]_o_ versus time for all dorsal striatum sites (sites #1–6). Average of 111–112 transients across six recording sites per genotype from five mice. **m** Mean peak ± SEM [DA]_o_ averaged across all dorsal striatum sites (sites #1–6). *n* = 5 mice per genotype. **p = 0.0087, paired two-tailed *t* test. **n** Mean ± SEM ratio of peak [DA]_o_ evoked by a four-pulse train to single-pulse stimulation in the dorsolateral striatum. *n* = 5 mice per genotype. **p = 0.0062, paired two-tailed *t* test. For **m**, **n**, dots represent values from individual mice. See also Supplementary Figs. 7 and 8. Source data are provided as a Source Data file.

To overcome the caveats of systemic rapamycin, we tested whether phenotypes in DA-Tsc1-KO mice could be prevented by cell type-specific genetic manipulation of mTORC1 signaling. To do this, we conditionally deleted one or two copies of the *Rptor* gene encoding the mTOR-binding protein Raptor in DA-Tsc1-KO mice to constrain mTORC1 signaling (Fig. 8a, b and Supplementary Fig. 7a). Raptor is an obligate member of mTORC1 and deletion of *Rptor* leads to selective mTORC1 loss of function^55,56^. This cell type-specific genetic strategy has two advantages over systemic pharmacologic suppression of mTOR: (1) manipulation of mTORC1 is restricted to DA neurons, and (2) mTORC1 activity can be selectively altered, without disrupting mTORC2.

We found that homozygous deletion of *Rptor* from DA-Tsc1-KO mice completely suppressed mTORC1 activity in DA neurons, as shown by the lack of p-S6 immunostaining (Supplementary Fig. 7b–f). DA neuron soma size was significantly smaller in DA-Tsc1-KO/Rptor-KO mice, and TH levels were reduced (Supplementary Fig. 7g, h). However, complete loss of Raptor was not able to rescue the dorsal striatal DA release deficits (Supplementary Fig. 7i–l). This may be a result of impaired DA synthesis, given the reduced TH levels. These results demonstrate that overactivation or complete suppression of mTORC1 are similarly detrimental to striatal DA transmission.

We next tested whether reduction, rather than complete suppression, of mTORC1 could prevent phenotypes in DA-Tsc1-KO mice. To do this, we compared DA-Tsc1-KO mice that were either wild-type or heterozygous (Het) for the conditional *Rptor* allele (Fig. 8b). Consistent with a reduction in mTORC1 signaling, p-S6 and TH levels in SNc neurons were significantly reduced in DA-Tsc1-KO/Rptor-Het mice compared to DA-Tsc1-KO/Rptor-WT (Fig. 8c–h). However, heterozygous deletion of *Rptor* did not prevent the increased soma size due to loss of Tsc1 (Fig. 8i). Therefore, heterozygous *Rptor* deletion constrained mTORC1 signaling in DA neurons in the context of Tsc1 loss, but was not sufficient to prevent somatic hypertrophy.

### Heterozygous *Rptor* deletion restores cognitive flexibility

To test whether the DA release deficits in DA-Tsc1-KO mice could be prevented by genetic reduction of mTORC1 signaling, we performed FCV experiments and found that DA-Tsc1-KO/Rptor-Het slices had significantly higher evoked [DA]_o_ throughout the dorsal striatum compared to DA-Tsc1-KO/Rptor-WT slices (Fig. 8j–m). Notably, DA release levels in DA-Tsc1-KO/Rptor-Het slices were comparable to levels in WT slices (Supplementary Fig. 8). We measured the 4*p*/1*p* ratio and found that DA-Tsc1-KO/Rptor-Het slices had reduced 4*p*/1*p* ratio compared to DA-Tsc1-KO/Rptor-WT slices, indicating higher *P*_r_ (Fig. 8n). Taken together, these results demonstrate that heterozygous *Rptor* deletion successfully relieved DA release deficits in the context of Tsc1 loss.

**Fig. 9:**
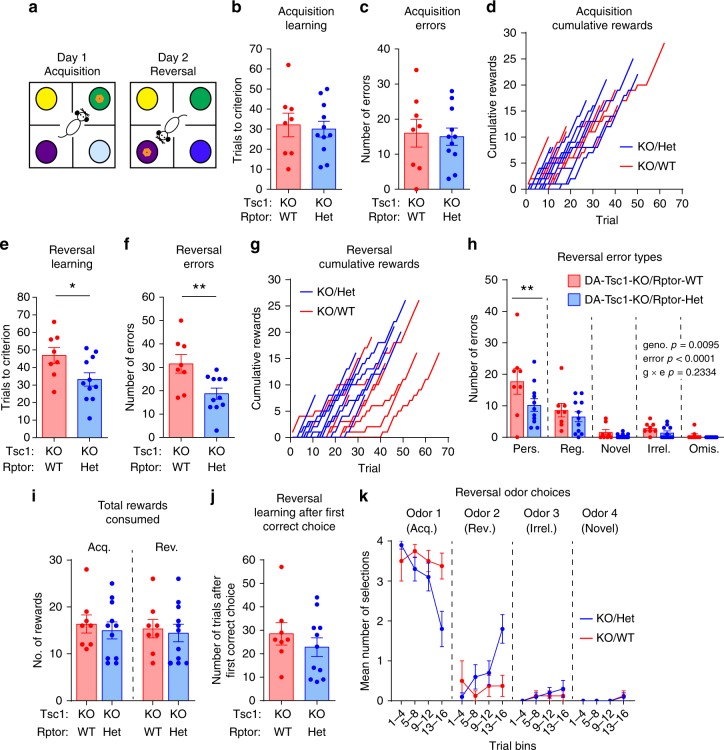
Heterozygous *Rptor* deletion increases cognitive flexibility in DA-Tsc1-KO mice. **a** Schematic of the four-choice odor-based reversal learning task. **b**, **c** Mean ± SEM number of trials taken to reach criterion (eight out of ten correct) during acquisition learning (**b**) and total errors made during acquisition learning (**c**) in the four-choice test. **d** Step function cumulative reward plot for individual animals during acquisition learning. **e,** **f** Mean ± SEM number of trials taken to reach criterion (eight out of ten correct) during reversal learning (**e**, *p = 0.0338) and total errors made during reversal learning (**f**, **p = 0.0095), unpaired, two-tailed *t* tests. **g** Step function cumulative reward plot for individual animals during reversal learning. **h** Analysis of different error types during reversal learning. Bars represent mean ± SEM number of perseverative (Pers.), regressive (Reg.), novel, irrelevant (Irrel.), or omission (Omis.) errors for mice of each genotype. Two-way ANOVA *p* values are shown. **p = 0.0072, Sidak’s multiple comparisons test. **i** Mean ± SEM total number of rewards obtained and consumed during acquisition (Acq.) and reversal (Rev.) learning. **j** Mean ± SEM and number of trials to reach criterion (eight out of ten correct) during reversal learning after the first correct selection of the newly rewarded odor. **k** Mean ± SEM number of odor selections in bins of four trials during reversal learning. Odor 1 is the odor rewarded during acquisition learning and odor 2 is the odor rewarded during reversal learning (**1** DA-Tsc1-KO/Rptor-Het mouse was excluded from this plot as it reached criterion in <16 trials). For all panels, DA-Tsc1-KO/Rptor-WT: *n* = 8 male mice and DA-Tsc1-KO/Rptor-Het: *n* = 11 male mice. For all bar graphs, dots represent values for individual mice. See Supplementary Table 5 for *p* values for all behavior tests. Source data are provided as a Source Data file.

Given the functional improvements in DA release, we tested whether DA-Tsc1-KO/Rptor-Het mice had increased cognitive flexibility in the four-choice reversal learning task (Fig. 9a). We observed no differences between male littermate DA-Tsc1-KO/Rptor-Het and DA-Tsc1-KO/Rptor-WT mice in acquisition learning (Fig. 9b–d). However, during reversal learning, DA-Tsc1-KO/Rptor-Het animals reached criterion in significantly fewer trials, made fewer total errors, and made fewer perseverative errors specifically (Fig. 9e–h). As discussed above for Fig. 7, these results are unlikely to be driven by major changes in odor preference or motivation (Fig. 9i, j). Plotting the odor choices across the first 16 trials of reversal learning showed that the DA-Tsc1-KO/Rptor-Het mice were faster to switch their odor choice (Fig. 9k). Together, these data demonstrate that cell type-specific genetic reduction of mTORC1 signaling in DA-Tsc1-KO mice improves evoked DA release and cognitive flexibility.

## Discussion

In this study, we used a cell type-targeted approach to investigate how loss of Tsc1 and deregulation of mTORC1 affect the dopaminergic system. We found that deletion of *Tsc1* from DA neurons strongly impacted their properties leading to somatodendritic and axonal hypertrophy, reduced intrinsic excitability, and impaired striatal DA release. These cellular changes were sufficient to reduce cognitive flexibility in a reversal learning task. We showed that DA release deficits and cognitive inflexibility in DA-Tsc1-KO mice could be prevented by genetic reduction of mTORC1 signaling. Together, these data provide insight into the potential cellular origins of cognitive inflexibility in TSC, and suggest a locus for possible intervention.

Several prior studies have assessed the consequences of deletion of the mTORC1-negative regulator *Pten* on DA neurons. Similar to loss of Tsc1, selective deletion of *Pten* from DA neurons causes somatodendritic hypertrophy, increased TH expression, and elevated total DA tissue content in the absence of changes to gross motor behavior^57–59^. However, distinct from *Pten* deletion^59^, we find that loss of Tsc1 is sufficient to dramatically increase DA axon terminal size in the dorsal striatum. Since we observed region-specific effects of Tsc1 loss on DA terminals, the differences may be due to the striatal region sampled for EM in prior studies, or may reflect distinct biological consequences of *Pten* versus *Tsc1* deletion. One notable difference is that *Pten* deletion increases both Akt and mTORC1 signaling^58,59^, whereas deletion of *Tsc1* activates mTORC1 but suppresses Akt phosphorylation^5^.

Our findings also show some similarities with prior work that deleted the macroautophagy regulator Atg7 from DA neurons. One of the key consequences of increased mTORC1 signaling is autophagy suppression^60^. Therefore, some of the Tsc1 phenotypes may result from impaired autophagy. Consistent with this, DA neuron-specific *Atg7*-KO mice also display increased soma size and axon terminal area^61^, suggesting that these phenotypes in DA-Tsc1-KO mice could be due to dysfunctional macroautophagy. That said, DA-Atg7-KO mice have increased striatal DA release and DA turnover^59,61^, which were not observed in DA-Tsc1-KO mice. Thus, while autophagy may be one downstream mechanism governing a subset of phenotypes in DA-Tsc1-KO neurons, there are likely additional cellular pathways that contribute to the overall complex phenotype resulting from Tsc1 loss.

We found that constitutive activation of mTORC1 signaling profoundly affected DA neuron structure and significantly impaired striatal DA release. This deficit was present in young adult mice and stably persisted in aged animals. We attempted to reverse these alterations with the widely used mTOR inhibitor rapamycin. However, we found unexpectedly that chronic systemic rapamycin treatment did not reduce p-S6 levels in DA neurons in either DA-Tsc1-WT or DA-Tsc1-KO mice. Since this lack of change was specific to DA neurons, with other brain regions showing the expected reduction in p-S6, we hypothesize that this could be due to two possibilities: (1) there is differential brain penetration of rapamycin, with midbrain DA neurons receiving lower effective concentrations and/or (2) there may be cell type-specific responses to systemic metabolic changes induced by rapamycin. For example, it is known that chronic rapamycin induces metabolic side effects, including glucose intolerance, fasting hyperglycemia and hyperinsulinemia due to actions on the liver and pancreas^62^. Regardless of the mechanism, these findings raise important caveats about the use of systemic rapamycin to treat the spectrum of neuropsychiatric phenotypes in TSC. We speculate that our findings provide a potential explanation for the failure of rapamycin derivatives to improve cognitive deficits in recent clinical trials in TSC^63,64^.

Given the challenges of systemic pharmacology, we utilized a genetic approach in which we selectively decreased mTORC1 signaling via deletion of the gene encoding the obligate mTORC1 protein Raptor in DA-Tsc1-KO mice. In addition to being cell type specific, this strategy avoids manipulation of mTORC2 signaling, which is known to occur with prolonged rapamycin treatment^65^. Interestingly, when we completely suppressed mTORC1 with homozygous deletion of *Rptor*, we found similar deficits in evoked DA release as with *Tsc1* deletion alone. The failure of complete mTORC1 suppression to prevent release deficits is consistent with prior reports showing that acute treatment of striatal slices with rapamycin or Cre virus-mediated deletion of *mTOR* from VTA neurons both decrease evoked DA release^61,66^. By contrast, we found that heterozygous loss of *Rptor* in DA-Tsc1-KO mice, while not sufficient to prevent somatic hypertrophy, significantly improved striatal DA release and countered reduced cognitive flexibility. Together, these results indicate that tight regulation of mTORC1 signaling is essential for DA neuron output as too much or too little mTORC1 signaling is similarly detrimental. In addition, our findings show that changes in DA neuron soma size can be decoupled from alterations in axonal DA release.

After ruling out several potential mechanisms for the release impairments in DA-Tsc1-KO mice, including DA neuron death, striatal denervation, and reduced DA synthesis, the most plausible explanation was ultrastructural changes in DA neuron axons. Specifically, we found that Tsc1 loss resulted in highly enlarged TH + axon terminals and reduced vesicle density in the striatum. In addition, the vesicles were further removed from the plasma membrane and were less tightly clustered together. Notably, axonal changes in the dorsal striatum, which is primarily innervated by SNc neurons, were much more pronounced than those in the NAc, which receives mainly VTA input. Given the stringent structural requirements for efficient vesicle release, for example, vesicle proximity to docking sites^67^ and vesicle clustering^68^, it is likely that the observed ultrastructural changes in DA-Tsc1 KO axons led to impaired dorsal striatal DA release with a relative sparing of NAc DA release.

Cognitive flexibility is commonly affected in psychiatric disorders, in particular ASD and ADHD^46,47,69^. TSC patients frequently present with impairments in executive control processes, which include cognitive flexibility^17,45^. Here, we found that DA neuron-specific deletion of *Tsc1* was sufficient to reduce cognitive flexibility in a reversal learning task. This reduction was specific to reversal learning as initial discrimination learning was intact. Notably, during reversal learning, DA-Tsc1-KO mice made more perseverative errors, in which they continued to return to the previously learned odor even though it was no longer rewarded. Perseverative errors reflect an inability to flexibly adapt and update a previously learned association. Such a deficit may contribute to the inflexible behaviors often observed in individuals with TSC^17^.

Our findings are consistent with prior reports showing that brain-wide heterozygous deletion of *Tsc2* or disruption of the translational regulator eIF4E, which increases protein synthesis, impair reversal but not acquisition learning in water maze tasks^70,71^. Our work suggests that these types of impairments may result from dopaminergic dysfunction; an interpretation consistent with the known involvement of DA in cognitive flexibility in rodents and humans^18,49^. Our results are also in agreement with studies showing that moderate reductions in dorsal striatal DA levels in rats can impair cognitive flexibility without changing motor behavior^72,73^. While our study demonstrates the sufficiency of dopaminergic impairments to induce cognitive inflexibility, alterations in other brain regions, including the cerebellum^13^, may also contribute to this complex phenotype in the context of TSC.

## Methods

### Mice

All animal procedures were carried out in accordance with protocols approved by the University of California, Berkeley Institutional Animal Care and Use Committee (IACUC). The ages of the animals used are indicated in the Method Details for each experiment. Unless otherwise indicated, mice of both sexes were used. Mice were an outbred strain on mixed genetic background as determined by the Charles River Mouse 384 SNP Complete Background Analysis Panel, with average percent heterozygosity of 22.9% (13.1–33.7% range). Mice were housed with same-sex littermates in groups of 5–6 animals per cage and kept on a regular 12 h light/dark cycle (lights on at 7:00 a.m.), with ad libitum access to food and water. Animals in behavior experiments were housed in a facility with a reverse 12 h light/dark cycle (lights off at 9:00 a.m.). Mice were switched to the reverse light cycle at least 2 weeks prior to behavior testing and were tested during the dark phase.

### Breeding strategy

To generate DA neuron-specific Tsc1-KO mice, *Tsc1*^*fl/fl*^ mice were bred to *DAT*^*IRES*^*Cre*^*wt/+*^ mice. To generate experimental animals, mice heterozygous for floxed *Tsc1* and *DAT*^*IRES*^*Cre* were crossed (*Tsc1*^*wt/fl*^*;DAT*^*IRES*^*Cre*^*wt/+*^ × *Tsc1*^*wt/fl*^*;DAT*^*IRES*^*Cre*^*wt/+*^). Experimental mice were heterozygous for Cre and either homozygous WT for Tsc1 (*Tsc1*^*wt/wt*^*;DAT*^*IRES*^*Cre*^*wt/+*^, referred to as DA-Tsc1-WT) or homozygous floxed for Tsc1 (*Tsc1*^*fl/fl*^*; DAT*^*IRES*^*Cre*^*wt/+*^, referred to as DA-Tsc1-KO).

For electrophysiology experiments, *Tsc1*^*fl/fl*^*;DAT*^*IRES*^*Cre*^*wt/+*^ mice were bred to the Ai9 tdTomato Cre-reporter line. *Tsc1*^*wt/fl*^*;DAT*^*IRES*^*Cre*^*wt/+*^*;Ai9*^*wt/+*^ × *Tsc1*^*wt/fl*^*;DAT*^*IRES*^*Cre*^*wt/+*^*;Ai9*^*wt/+*^ crosses were used to generate experimental mice. Mice used for experiments were either heterozygous or homozygous for the Ai9 transgene.

For genetic rescue experiments, *Tsc1*^*fl/fl*^*;DAT*^*IRES*^*Cre*^*wt/+*^ mice were crossed with *Rptor*^*fl/fl*^ mice. To generate experimental animals, a *Tsc1*^*fl/fl*^*;Rptor*^*wt/fl*^*;DAT*^*IRES*^*Cre*^*wt/+*^ × *Tsc1*^*fl/fl*^*;Rptor*^*wt/fl*^*;DAT*^*IRES*^*Cre*^*wt/+*^ breeding strategy was used. The resulting offspring were all homozygous floxed for *Tsc1* and either wild type (*Tsc1*^*fl/fl*^*;Rptor*^*wt/wt*^*;DAT*^*IRES*^*Cre*^*wt/+*^, referred to as DA-Tsc1-KO/Rptor-WT), heterozygous (*Tsc1*^*fl/fl*^*;Rptor*^*wt/fl*^*;DAT*^*IRES*^*Cre*^*wt/+*^, referred to as DA-Tsc1-KO/Rptor-Het), or homozygous floxed (*Tsc1*^*fl/fl*^*;Rptor*^*fl/fl*^*;DAT*^*IRES*^*Cre*^*wt/+*^, referred to as DA-Tsc1-KO/Rptor-KO) for *Rptor*.

To compare *Tsc1*^*fl/fl*^*;Rptor*^*wt/fl*^*;DAT*^*IRES*^*Cre*^*wt/+*^ mice with *Tsc1*^*wt/wt*^*;Rptor*^*wt/wt*^*;DAT*^*IRES*^*Cre*^*wt/+*^ mice, a *Tsc1*^*wt/fl*^*;Rptor*^*wt/fl*^*;DAT*^*IRES*^*Cre*^*wt/+*^ × *Tsc1*^*wt/fl*^*;Rptor*^*wt/fl*^*;DAT*^*IRES*^*Cre*^*wt/+*^ cross was made. Offspring wild type for *Tsc1* and *Rptor (Tsc1*^*wt/wt*^*;Rptor*^*wt/wt*^*;DAT*^*IRES*^*Cre*^*wt/+*^, referred to as DA-Tsc1-WT/Rptor-WT) were compared to DA-Tsc1-KO/Rptor-Het (*Tsc1*^*fl/fl*^*;Rptor*^*wt/fl*^*;DAT*^*IRES*^*Cre*^*wt/+*^, referred to as *DA-Tsc1-KO/Rptor-Het)* animals.

See Supplementary Table 7 for a list of the transgenic mouse lines used and Supplementary Table 8 for a list of mouse genotyping primers.

### Electrophysiology

Male and female adult mice (P56–P80) were deeply anesthetized by isoflurane, transcardially perfused with ice-cold high Mg^2+^ artificial cerebrospinal fluid (aCSF) using a peristaltic pump (Instech) and decapitated. Coronal midbrain slices (275 μm thick) were prepared on a vibratome (Leica VT1000 S) in ice-cold high Mg^2+^ aCSF containing (in mM): 85 NaCl, 25 NaHCO_3_, 2.5 KCl, 1.25 NaH_2_PO_4_, 0.5 CaCl_2_, 7 MgCl_2_, 10 glucose, and 65 sucrose. Slices were recovered for 15 min at 34 °C, followed by at least 50 min at room temperature (RT) in aCSF containing (in mM): 130 NaCl, 25 NaHCO_3_, 2.5 KCl, 1.25 NaH_2_PO_4_, 2 CaCl_2_, 2 MgCl_2_, and 10 glucose. All solutions were continuously bubbled with 95% O_2_ and 5% CO_2_.

Recordings were performed at 32 °C in the presence of AMPA (α-amino-3-hydroxy-5-methyl-4-isoxazolepropionic acid), NMDA (*N*-methyl-d-aspartate), and GABA_A_ (type A γ-aminobutyric acid) synaptic blockers (10 μM GYKI 52466; 10 μM CPP; 50 μM picrotoxin, final concentration, all from Tocris), with a bath perfusion rate of ~2 ml/min. Dopaminergic neurons in the SNc and VTA were identified by Ai9 tdTomato fluorescence. For whole-cell recordings, 2.5–6 mΩ borosilicate glass pipettes (Sutter Instruments: BF150-86-7.5) were filled with a potassium-based internal solution containing (in mM): 135 KMeSO_3_, 5 KCl, 5 HEPES, 4 Mg-ATP, 0.3 Na-GTP, 10 phospho-creatine, 1 EGTA, and 4 mg/ml neurobiotin (Vector Laboratories #SP-1120). Recordings were obtained using a MultiClamp 700B amplifier (Molecular Devices) and ScanImage software (https://github.com/bernardosabatini/SabalabAcq). Passive membrane properties were recorded in voltage clamp with the membrane held at −70 mV. Negative current steps (2 s, −50 to −200 pA) were applied to measure the sag amplitude and rebound following hyperpolarization. Positive current steps (2 s, +25 to +600 pA) were applied to generate an input–output curve from a baseline membrane potential of −70 mV as maintained by current clamp. For whole-cell recordings, series resistance was <30 MΩ and liquid junction potential was not corrected.

Electrophysiology data were acquired using the ScanImage software, written and maintained by Dr. Bernardo Sabatini (https://github.com/bernardosabatini/SabalabAcq). Data were analyzed in Igor Pro (Wavemetrics). Evoked AP properties were measured from the first AP in the train of evoked APs. AP threshold was calculated by taking the second derivative of the AP. AP duration was measured at the half-maximal membrane potential. Rheobase was calculated as the current at which APs were first elicited. Passive properties were calculated from an RC check in voltage clamp recordings at −70 mV. Passive properties and AP properties were analyzed for significance using two-tailed unpaired *t* tests, Mann–Whitney tests, or *t* tests with Welch’s correction. Excitability curves were analyzed for significance using a two-way analysis of variance (ANOVA) with Sidak’s multiple comparison tests.

### Neurobiotin-filled neuron reconstruction

DA neurons were filled with neurobiotin-containing internal solution (4 mg/ml) during whole-cell recordings. Slices were fixed in 4% paraformaldehyde solution (Electron Microscopy Sciences: 15713) in 1× phosphate-buffered saline (PBS) for 24–48 h at 4 °C. With continuous gentle shaking, slices were washed in 1× PBS 3 × 5 min and incubated with BlockAid blocking solution (Life Tech: B10710) for 1 h at room temperature (RT). Primary antibodies against TH (1:1000, Immunostar: 22941) and streptavidin–Alexa Flour 488 conjugate (1:750, Invitrogen: S11223) were applied overnight at 4 °C in 1× PBS containing 0.25% Triton X-100 (PBS-Tx). The following day, slices were washed in 1× PBS 3 × 5 min, and secondary Alexa-633 goat anti-mouse antibody (1:500, Thermo Fisher: A21050) in PBS-Tx was applied for 1 h at RT. Slices were washed in cold 1× PBS 5 × 5 min, mounted on SuperFrost slides (VWR: 48311-703) with the neurobiotin-filled cell facing up, and coverslipped with either Prolong Gold antifade (Life Tech: P36935) or Vectashield hard-set (Vector Labs: H-1500) mounting media.

Neurobiotin-labeled cells were imaged on a Zeiss LSM 880 NLO AxioExaminer confocal with ×20/1.0 NA water-immersion objective and 488 Argon laser using 1.53 µm steps to acquire a z-stack image spanning the entirety of the neurobiotin-filled cell body and dendritic arbor. 3D reconstruction of the cells was performed using the IMARIS software (Bitplane) with automated filament tracing and manual editing. The mask generated by the automated filament tracing algorithm was continuously cross-referenced with the original z-stack image to ensure accuracy. Spurious segments created by the automated filament tracer were removed, while processes with incomplete reconstruction were edited to incorporate missing segments. Sholl analysis of dendritic arborization was performed by quantifying the number of intersections of dendrites with concentric circles drawn at 1 µm steps, starting 7 µm from the center point of the soma.

Total dendrite length was analyzed using an unpaired *t* test and Sholl intersections were analyzed using a repeated-measures two-way ANOVA.

### Fast-scan cyclic voltammetry (FCV)

DA release was monitored using FCV in acute coronal slices^36,74,75^. Paired site-sampling interleaved recordings were performed in slices from one reference and one experimental animal in a sex- and age-matched mouse pair recorded on the same day using the same carbon fiber microelectrode (CFM). The genotype order of tissue prep was counterbalanced between experiments. Male and female mice (P56-P95 or P660-725) were deeply anesthetized by isoflurane and decapitated. 275 μm thick coronal striatal slices were prepared on a vibratome (Leica VT1000 S) in ice-cold high Mg^2+^ ACSF containing in mM: 85 NaCl, 25 NaHCO_3_, 2.5 KCl, 1.25 NaH_2_PO_4_, 0.5 CaCl_2_, 7 MgCl_2_, 10 glucose, and 65 sucrose. Slices were recovered for 1 h at RT and were recorded from in ACSF containing in mM: 130 NaCl, 25 NaHCO_3_, 2.5 KCl, 1.25 NaH_2_PO_4_, 2 CaCl_2_, 2 MgCl_2_, and 10 glucose. All solutions were continuously saturated with 95% O_2_ and 5% CO_2._ Slices between +1.5 mm and +0.5 mm from Bregma containing dorsal striatum and NaC (ventral striatum) were used for experimentation^76^.

In the recording chamber, slices were maintained at 32 °C with a superfusion rate of 1.2–1.4 ml/min. Extracellular DA concentration ([DA]_o_) was monitored with FCV at CFMs using a Millar voltammeter (Julian Millar, Barts and the London School of Medicine and Dentistry). CFMs were fabricated in-house from epoxy-free carbon fiber ~7 μm in diameter (Goodfellow Cambridge Ltd) encased in a glass capillary (Harvard Apparatus: GC200F-10) pulled to form a seal with the fiber and cut to a final tip length of 70–120 μm. The CFM was positioned ~100 μm below the tissue surface at a 45° angle. A triangular waveform was applied to the carbon fiber scanning from −0.7 to +1.3 V and back, against a Ag/AgCl reference electrode at a rate of 800 V/s. Evoked DA transients were sampled at 8 Hz, and data were acquired at 50 kHz using AxoScope 10.5 (Molecular Devices). Oxidation currents evoked by electrical stimulation were converted to [DA]_o_ from post-experimental calibrations of the CFM. Recorded FCV signals were identified as DA by comparing oxidation (+0.6 V) and reduction (−0.2 V) potential peaks from experimental voltammograms with currents recorded during calibration with 2 μM DA dissolved in aCSF and all drug-containing media used in a given experiment.

FCV data were pre-processed using the AxoScope 10.5 (Molecular Devices) software and analyzed using Excel. Within a given experiment, quinpirole data were normalized to control drug-free data, frequency data were normalized to single pulses, and current-amplitude data were normalized to 1200 μA current data, before collating across experiments. We included a minimum of three release events for each stimulus or condition at each individual recording site for these experiments. Quinpirole effects were compared using sigmoidal curve-fit analyses. Current-amplitude and frequency-response data were compared using a two-way ANOVA with Sidak’s multiple comparisons tests.

For all site-sampling experiments (aCSF, DHβE, oxo-M, 5 mM Ca^2+^) single pulse and four pulses at 100-Hz-evoked DA concentrations were compared without normalization because control and experimental slices were recorded with the same CFM for every experimental pair. A minimum of four matched mouse pairs were used in these experiments, with two slices per animal recorded from. Single-pulse data includes two release events per site per slice, and pulse train data includes one release event per site per slice. Peak-evoked DA release levels were compared between genotypes using paired *t* test tests or Wilcoxon’s *t* tests.

Extracellular DA uptake is exponentially related to the substrate concentration and DAT expression can vary by striatal region^32,40^. To meaningfully compare DAT kinetics, we identified concentration- and striatal region-matched transients from DA-Tsc1-WT and DA-Tsc1-KO slices and examined their decay kinetics using one-phase exponential decay curve-fit analyses. Only the data from single-pulse stimulations were used for these analyses.

### FCV electrical stimulation and drug application

Following 30 min slice equilibration in the recording chamber, DA release was evoked using square wave pulses (0.6 mA pulse amplitude, 2 ms pulse duration) controlled by a Master-8 pulse stimulator or Isoflex stimulus isolator (A.M.P.I., Jerusalem, Israel) delivered out of phase with voltammetric scans. A concentric bipolar stimulating electrode (FHC: CBAEC75) used for electrical stimulation was positioned on the slice surface first with minimal tissue disturbance, followed by CFM inserted 100 μm away from the bipolar electrode. Three stimulation paradigms were used: single stimulation pulse, paired single pulses delivered 3 s apart, and brief four-pulse stimulation trains delivered at 5, 10, 25, 40, or 100 Hz. Stimuli were delivered every 2.5 min.

For site-sampling experiments, three stimulations were delivered at a given site before moving to the corresponding site in the slice from the paired mouse. Stimulations were delivered in the following order: single pulse, pulse train of four pulses at 100 Hz, single pulse. The sampling sites were: (1) dorsolateral striatum, (2) dorsocentral striatum, (3) dorsomedial striatum, (4) central striatum, (5) ventrolateral striatum, (6) ventromedial striatum, and (7) NAc core (two sampling sites within the core were averaged together for quantification).

For quinpirole experiments, recordings were performed from a single site per slice. After reaching a steady baseline release with single pulses in control aCSF, increasing doses of quinpirole (1 nM–10 µM, Tocris) were bath-applied for ~10 min each, with steady-state release achieved for at least four consecutive stimulations before progression to the next highest drug concentration. Traces are peak normalized to control values prior to drug application within each genotype for quantification.

For current-amplitude and frequency-response recordings, recordings were performed from a single site per slice. After reaching a steady baseline release with single pulses, each stimulation condition was repeated three times at each frequency/current amplitude in a pseudorandom order.

### Immunohistochemistry

Male and female mice (P75–P120) were deeply anesthetized by isoflurane and exsanguinated by transcardial perfusion with ice-cold 1× PBS (~5–7 ml), followed by 4% paraformaldehyde (PFA) solution (Electron Microscopy Sciences: 15713) in 1× PBS (~5–10 ml) using a peristaltic pump (Instech). The brains were removed and post fixed by immersion in 4% PFA in 1× PBS overnight at 4 °C. Brains were then suspended in 30% sucrose in 0.1 M PB solution for cryoprotection. After brains descended to the bottom of the vial (typically 24–28 h), 30 μm coronal sections of the midbrain or striatum were cut on a freezing microtome (American Optical AO 860), collected into serial wells, and stored at 4 °C in 1× PBS containing 0.02% (w/v) sodium azide (NaN_3_; Sigma-Aldrich).

Free-floating brain sections for immunohistochemistry were batch processed to include matched control and experimental animals. With gentle shaking, sections were washed 3 × 5 min in 1× PBS, followed by 1 h incubation at RT with BlockAid blocking solution (Life Tech: B10710). Primary antibodies were applied at 4 °C in PBS-Tx for 48–72 h. Sections were washed with cold 1× PBS 3 × 5 min, incubated for 1 h at RT with secondary antibodies in PBS-Tx, washed in cold 1× PBS 5 × 5 min, mounted on SuperFrost slides (VWR: 48311-703), and coverslipped with Vectashield hard-set (Vector Labs: H-1500) mounting media. For DA neuron count experiments, slices underwent no additional processing apart from 1× PBS wash 5 × 5 min. Experimenters were blind to animals’ genotype throughout tissue processing and data analysis. See Supplementary Table 9 for a list of antibodies used for immunohistochemistry.

### Confocal microscopy

Images of brain sections were acquired using a Zeiss LSM 710 AxioObserver confocal microscope fitted with a motorized XY stage for tile scanning. A ×20/0.8 NA air objective was used to generate tile scans (424 × 424 μm per tile) of one hemisphere (5 × 3 grid) or the entire midbrain (10 × 3 grid). The 405 nm, 488 nm, 561 nm, and 633 nm lasers were used. Z-stack images captured the entire thickness of the slice at 1.10–1.25 μm steps. Laser power settings and acquisition parameters were kept constant for all experimental conditions.

Image analysis was performed blind to genotype. Quantification was performed on max-projected z-stack images, two sections per mouse were analyzed and averaged together. For soma size and p-S6 measurements, soma boundaries were traced manually using the Image J (NIH) software in the TH channel to include all clearly identifiable single cells in the SNc or VTA (minimum of 100 cells for each area in each slice). Soma cross-sectional area and fluorescence intensity for the p-S6 channel were extracted automatically in Image J. Cumulative distributions were analyzed using Kolmogorov–Smirnov tests (when comparing two groups) or the Kruskal–Wallis test with Dunn’s multiple comparisons tests (when comparing four groups).

For DA neuron counts, every clearly identifiable tdTomato + cell in both hemispheres in both the SNc and VTA was manually counted using the Cell Counter Image J (NIH) plug-in, and analyzed using an unpaired *t* test. Three anatomically matched sections in the rostro-caudal plane per mouse were counted in three age- and sex-matched mouse pairs (i.e. nine sections were counted from three mice per genotype).

### High-performance liquid chromatography

Tissue DA content was measured by HPLC with electrochemical detection in tissue punches from dorsal and ventral striatum. Male and female mice (P60–P75) were deeply anesthetized by isoflurane and decapitated. Coronal slices (300  μm thick) of striatum were prepared on a vibratome (Leica VT1000 S) in ice-cold high Mg^2+^ aCSF containing (in mM): 85 NaCl, 25 NaHCO_3_, 2.5 KCl, 1.25 NaH_2_PO_4_, 0.5 CaCl_2_, 7 MgCl_2_, 10 glucose, and 65 sucrose. Slices were recovered for 1 h at RT in aCSF containing (in mM): 130 NaCl, 25 NaHCO_3_, 2.5 KCl, 1.25 NaH_2_PO_4_, 2 CaCl_2_, 2 MgCl_2_, and 10 glucose. All solutions were continuously bubbled with 95% O_2_ and 5% CO_2_. Following slice recovery, tissue punches from the dorsal striatum (2.5 mm diameter, aligned to the edge of the corpus callosum on the dorsolateral side) and ventral striatum (1.5 mm diameter, centered on the anterior commissure) from two brain slices per animal were taken and stored at −80 °C in 200 µl of 0.1 M HClO_4_. Ventral striatum samples hence included both NAc core and shell. On the day of analysis, samples were thawed, homogenized, and centrifuged at 16,000 × *g* for 15 min at 4 °C. The supernatant was analyzed for DA, DOPAC, and 5-hydroxyindoleacetic acid content using HPLC with electrochemical detection. Analytes were separated using a 4.6 × 150 mm^2^ Microsorb C18 reverse-phase column (Varian or Agilent) and detected using a Decade II SDS electrochemical detector with a Glassy carbon working electrode (Antec Leyden) set at +0.7 V with respect to a Ag/AgCl reference electrode. The mobile phase consisted of 13% methanol (v/v), 0.12 M NaH_2_PO_4_, 0.5 mM OSA (osanetant), 0.8 mM EDTA, pH 4.8, and the flow rate was fixed at 1 ml/min. Analyte measurements were normalized to tissue punch volume (pmol/mm^3^). HPLC data was collected with Clarity (DataApex, release version 2.6). HPLC analysis was repeated in two independent experiments.

Analysis was performed blind to genotype. Data were normalized and expressed as a percent of control within a given experiment before collating across experiments (samples were collected and analyzed in two batches). Two samples were analyzed per mouse and averaged together. Data were analyzed for statistical significance using a two-tailed unpaired *t* test.

### Western blotting

Male and female mice (P60–P90) were deeply anesthetized by isoflurane and decapitated. Bilateral striata, including both dorsal and ventral regions, were rapidly dissected on ice, flash-frozen in liquid nitrogen, and stored at −80 °C. On the day of analysis, frozen samples were sonicated until homogenized (QSonica Q55) in 500 μl lysis buffer containing 1% SDS in 1× PBS with Halt phosphatase inhibitor cocktail (Fisher: PI78420) and Complete mini EDTA-free protease inhibitor cocktail (Roche: 4693159001). Sample homogenates were then boiled on a heat block at 95 °C for 10 min, allowed to cool down to RT, and total protein content was determined by BCA assay (Fisher: PI23227). Following BCA assay, protein homogenates were mixed with 4× Laemmli sample buffer (Bio-Rad: 161-0747). Proteins (10–15  μg) were loaded onto 4–15% Criterion TGX gels (Bio-Rad: 5671084). Proteins were transferred to PVDF membrane (Bio-Rad: 1620177) at 4 °C overnight using the Bio-Rad Criterion Blotter (12 V constant voltage). The membranes were blocked in 5% milk in 1× TBS with 1% Tween (TBS-T) for 1 h at RT, and incubated with primary antibodies diluted in 5% milk in TBS-T overnight at 4 °C. The following day, after 3 × 10 min washes with TBS-T, the membranes were incubated with horseradish peroxidase-conjugated secondary antibodies for 1 h at RT. Following 6 × 10 min washes, the membranes were incubated with chemiluminesence substrate (PerkinElmer: NEL105001EA) for 1 min and exposed to GE Amersham Hyperfilm ECL (VWR: 95017-661). Membranes were stripped with re-blot plus strong solution (Millipore: 2504) to re-blot on subsequent days.

Western blot analysis was performed blind to genotype. Bands were quantified by densitometry using the Image J (NIH) software. The striatal-enriched protein DARPP-32 was used to normalize striatal tissue content and histone 3 was used as a loading control. Samples were taken from the whole striatum, including both dorsal and ventral regions. Two striatum samples (i.e. left and right hemispheres) were analyzed per mouse and averaged together. Once normalized to DARPP-32, protein content was expressed as percentage of control within a given experiment. TH data were analyzed using an unpaired *t* test with Welch’s correction and VMAT-2 data were analyzed using a Mann–Whitney test. Uncropped western blots are provided in the Source Data file. See Supplementary Table 10 for a list of antibodies used for western blotting.

### Sample preparation for EM

Male and female mice (P75–P85) were deeply anesthetized by isoflurane, exsanguinated by transcardial perfusion with ice-cold 0.1 M PB (pH 7.4), followed by fixation with 0.1% glutaraldehyde (Sigma-Aldrich: G5882) and 4% paraformaldehyde (Electron Microscopy Sciences: 15713) solution in 0.1 M PB. Perfusions were performed using a peristaltic pump (Instech). The brains were removed and immersed in the fixative solution for an additional 12 h at 4 °C. Brains were then stored in 0.1 M PB with 0.02% (w/v) NaN_3_. Coronal sections (50 µm) containing dorsal striatum and/or NAc core were cut using a vibrating-blade microtome (Leica VT1000 S). Tissue sections for analyses of dorsal striatum were promptly processed for immunolabeling. Tissue sections for analyses of NAc were stored in 1x PBS with NaN_3_ at 4 °C for processing at a later date. To start the preparation of tissue for immunolabeling, sections were washed 3 × 5 min in 1× PBS, placed into cryoprotectant (0.05 M PB, 25% sucrose, 10% glycerol) for a minimum of 2 h and then freeze-thawed three times in liquid nitrogen to increase penetration of the reagents. Sections were washed thoroughly and blocked with 10% normal goat serum in 1× PBS (Vector Laboratories: S-1000) for 1 h, followed by overnight incubation in primary rabbit anti-TH antibody (1:1000, Chemicon: #AB152) at RT. Sections were washed 3 × 5 min in 1× PBS and incubated in a gold-conjugated goat anti-rabbit secondary antibody (1:400, 1.4 nm Nanogold, Nanoprobes: #2003) for a minimum of 4 h at RT. TH-positive axons were revealed with silver intensification of the conjugated gold particles. Silver reagent (1 ml; Nanoprobes: HQ Silver Kit, prepared according to the manufacturer’s instructions) was added to each section and allowed to react for 4.5 min in the dark, washed 3 × 5 min in acetate buffer (0.1 M sodium acetate 3-hydrate, pH 7.0–7.5), and then 1× PBS for 5 min. All sections were then washed 3 × 5 min in 0.1 M PB. The sections were post fixed in 1% osmium tetroxide in 0.1 M PB (OsO_4_; TAAB) for 12 min. After washing 2 × 5 min in 0.1 M PB, sections were dehydrated in an ascending series of ethanol dilutions: 2 × 10 min in 50% ethanol; 45–60 min in 70% ethanol, which included 1% uranyl acetate (TAAB); 10 min in 95% ethanol; and 2 × 10 min in absolute ethanol. Sections were washed 2 × 10 min in propylene oxide (Sigma-Aldrich), placed into resin (Durcupan ACM; Fluka), and left overnight (~15 h) at RT. The resin was then warmed to reduce its viscosity, and sections were placed on microscope slides, coverslipped, and the resin cured at 65 °C for ~70 h^77,78^. Experimenters were blind to genotype throughout tissue processing and data analysis.

### Electron microscopy

All sections were examined under a light microscope to select those containing striatal areas that were similar to those sampled in FCV recordings. The selected areas of dorsolateral striatum and NAc core were cut from the slide, glued to the top of a resin block, and trimmed with razor blades. For each mouse, dorsal striatal regions from two sections across the rostro-caudal plane were examined; for the NAc, the core region was examined from one section. Serial sections, ~50 nm thick, were cut using an ultramicrotome (Leica EM UC6), collected on pioloform-coated, single-slot copper grids (Agar Scientific), and lead stained with Reynold’s lead citrate to improve contrast for electron microscopic examination. For each region (block), ultrathin sections from at least two grids were examined. A Philips CM100 or a Hitachi HT7800 transmission electron microscope was used to examine the ultrathin sections of dorsal striatum or NAc, respectively. Analyses of pre-embedded immunogold sections were performed at a minimum of 5 µm from the tissue–resin border (i.e., section surface). The maximum distance from the tissue–resin border examined was determined by the penetration of the gold-conjugated antibody together with the angle at which the tissue–resin was sectioned, and was therefore variable. TH-positive structures were systematically analyzed in one of the serial sections on an electron microscopic grid. At a magnification at which it is not possible to clearly visualize synapses, an area was chosen at random, the magnification was then increased and the first structure positively labeled for TH was digitally recorded (with either a GatanCCD UltraScan US1000 camera [Gatan] fitted to the Philips CM100, or with a XAROSA CMOS camera [Emsis] fitted to the Hitachi HT7800). TH-positive structures were identified and imaged, in this way, continuing systematically in straight lines across the section, keeping the identified TH-positive structure central within the image frame. For immunogold-labeled structures, the criterion for an immunopositive structure was five or more silver-intensified immunogold particles. For dorsolateral striatum, this was repeated for the second block until a minimum of 60 TH-immunopositive structures were identified and imaged per animal (491 dopaminergic profiles in total, *n* = 4 mice per genotype). For the NAc core, 30 profiles were imaged per block (240 profiles in total, *n* = 4 mice per genotype). Symmetric synapses (Gray’s type II) were identified by the presence of presynaptic and postsynaptic membrane specializations, a widened synaptic cleft and cleft material. Any TH-positive structures seen to be forming such symmetric synapses were imaged^68,79^. Electron micrographs were acquired using the Digital Micrograph (GATAN, release version 3) software for dorsal striatum and the Radius (EMSIS, release version 2) software for NAc core.

EM analysis was performed blind to genotype. Digital images were analyzed using Image J (NIH) and the Image J plug-ins PointDensity and PointDensitySyn (https://liu.se/medfak/forskning/larsson-max/software?l=en; ref. ^80^). Images were adjusted for contrast and brightness using Photoshop (version CS5; Adobe Systems). For analysis of TH-positive structures, the central TH-positive structure within the image frame was analyzed by tracing the perimeter. Other TH-positive structures completely within the electron micrograph frame were also analyzed. Analysis of immunogold-labeled TH-positive structures was performed using PointDensity; after the perimeter of the central structure within the frame was delineated, a point marker was placed within the center of each vesicle. Vesicles were marked if at least 50% of the vesicle membrane was visible. If a structure was observed to form a synaptic specialization, it was reanalyzed separately, and the length of the active zone(s) was delineated and measured. The active zone was defined as the length of plasma membrane directly opposing the postsynaptic density, across the synaptic cleft. TH-positive profiles that were forming synapses were analyzed separately using PointDensitySyn; the plasma membrane of the structure was traced, the active zone(s) were delineated, and points were placed in the center of the vesicles^68,79^. Cumulative distributions for EM data were analyzed using Kolmogorov–Smirnov tests. Inter-vesicle distance data binned at 50 nm intervals for dorsal striatum or 70 nm for NAc was analyzed using a two-way ANOVA with Sidak’s multiple comparisons tests.

To confirm that the increased sampling of dorsal striatum profiles compared to NAc profiles did not affect the statistical conclusions, we down-sampled the dorsal striatum data to match the NAc data by randomly taking 30 profiles per mouse, for a total of 120 profiles per genotype. The down-sampled data was highly overlapping with the original full data set and genotype comparisons of profile perimeter, area, number of vesicles, and vesicle density remained significantly changed with a *p* value of <0.0001 (Kolmogorov–Smirnov test).

### Behavior testing

Behavior testing was carried out in the dark phase of the light cycle under red lights, except the rotarod and four-choice reversal tests, which were carried out in the dark phase under white lights. Mice were habituated to the behavior room for at least 30 min prior to testing and covered by a black-out curtain. Mice were given at least one day in between different tests. All behavior equipment was cleaned between each trial/mouse with 70% ethanol, and additionally rinsed in diluted soap followed by water at the end of the day. If male and female mice were to be tested on the same day, male mice were tested first, returned to the husbandry room, after which all equipment was thoroughly cleaned, and female mice were brought in for habituation. Behavioral tests were performed with P60–80 male and female mice, except for the four-choice test for which P70–130 male animals were used.

The experimenter was blind to genotype throughout the testing and scoring procedures. Datasets with non-normal distributions were analyzed using a Mann–Whitney test, datasets with a normal distribution were analyzed using two-tailed unpaired *t* tests. Rotarod and four-choice reversal test were analyzed using a two-way ANOVA with Sidak’s multiple comparisons tests. The three-chamber social approach test was analyzed within genotype to test for significant differences between mouse and object preference with paired *t* tests.

### Four-choice odor-based reversal task

The four-choice odor-based reversal task^53^ was used to assess learning and cognitive flexibility in adult male mice. Animals were food restricted for 5–6 days in total with unrestricted access to drinking water, and maintained at 85–90% of ad lib feeding body weight. Food was given at the end of the day once testing was completed. Food restriction began 24 h before pre-training.

The four-choice test was performed in a custom-made square box (30.5 cm L × 30.5 cm W × 23 cm H) constructed of clear acrylic. Four internal walls 7.6 cm wide partially divided the arena into four quadrants. A 15.2 cm diameter removable cylinder fit in the center of the maze and was lowered between trials (after a digging response) to isolate the mouse from the rest of the maze. Odor stimuli were presented in white ceramic pots measuring 7.3 cm diameter and 4.5 cm deep. Pots were sham baited with a cereal reward, either a Honey Nut Cheerio (General Mills, Minneapolis, MN) or Fruit Loop cereal (Kellogg’s, Battle Creek, MI) secured underneath a mesh screen at the bottom. The apparatus was cleaned with 2.5% acetic acid followed by water between mice. Pots were cleaned with 70% ethanol followed by water. The apparatus was cleaned with diluted soap and water at the end of each testing day.

On the first habituation day of pre-training (day 1), animals were allowed to freely explore the testing arena for 30 min and consume small pieces of Cheerios placed inside empty pots positioned in each of the four corners. On the second shaping day of pre-training (day 2), mice learned to dig to find cereal pieces buried in unscented coarse pine wood shavings (Harts Mountain Corporation, Secaucus, NJ). On the shaping day, animals that failed to consume Cheerio pieces were switched to Fruit Loops, which they consumed successfully. A single pot was used and increasing amounts of unscented wood shavings were used to cover each subsequent cereal reward. The quadrant containing the pot was alternated on each trial and all quadrants were rewarded equally. Trials were untimed and consisted of (in order): two trials with no shavings, two trials with a dusting of shavings, two trials with the pot a quarter full, two trials with the pot half full, and four trials with the cereal piece completely buried by shavings. The mouse was manually returned to the center cylinder between trials.

On the days for odor discrimination (day 3, aquisition) and reversal (day 4), wood shavings were freshly scented on the day of testing (Table 1). Anise extract (McCormick, Hunt Valley, MD) was used undiluted at 0.02 ml/g of shavings. Clove, litsea, and eucalyptus oils (San Francisco Massage Supply Co., San Francisco, CA) were diluted 1:10 in mineral oil and mixed at 0.02 ml/g of shavings. Thymol (thyme; Alfa Aesar) was diluted 1:20 in 50% ethanol and mixed at 0.01 ml/g of shavings. During the discrimination phase (day 3), mice had to discriminate between four pots with four different odors and learn which one contained a buried food reward (footnote a indicates the rewarded odor in the table below). Each trial began with the mouse confined to the start cylinder, once the cylinder was lifted, timing began and the mouse could freely explore the arena until it chose to dig in a pot. Digging was defined as purposefully moving the shavings with both front paws. A trial was terminated if no choice was made within 3 min and recorded as omission. Criterion was met when the animal completed eight out of ten consecutive trials correctly.

**Table 1:** Odors used during acquisition and reversal learning in the four-choice test.

	Odor 1	Odor 2	Odor 3	Odor 4
Acquisition	^a^Anise	Clove	Litsea	Thyme
Reversal	Anise	^a^Clove	Litsea	Eucalyptus

^a^The odor rewarded on each testing day

The reversal phase of the task was completed the following day after overnight consolidation (day 4). Mice first performed the task with the same rewarded odor as the discrimination day to ensure they learned and remembered the task. After reaching criterion on recall (eight out of ten consecutive trials correct), the rewarded odor was switched and mice undergo a reversal learning phase in which a previously unrewarded odor is rewarded. Perseverative errors were choices to dig in the previously rewarded odor that was no longer rewarded. Regressive errors were choosing the previously rewarded odor after the first correct choice of the newly rewarded odor. Novel errors were choices to dig in the pot with the newly introduced odor for reversal testing. Irrelevant errors were choices to dig in the pot that had never been rewarded. Omissions were trials in which the mouse failed to make a digging choice within 3 min from the start of the trial. Total errors are the sum of perseverative, regressive, irrelevant, novel, and omission errors. Criterion was met when the mouse completed eight out of ten consecutive trials correctly. The spatial location of the odors was shuffled on each trial.

### Open-field test

Exploratory behavior in a novel environment and general locomotor activity were assessed by a 60-min session in an open-field chamber (40 cm L × 40 cm W × 34 cm H) made of transparent plexiglas. Horizontal photobeams to measure rearing were positioned at 9 cm height from the floor. The mouse was placed in the bottom right hand corner of the arena and behavior was recorded using an overhead camera and analyzed using the ANY-maze (Stoelting Co.) behavior tracking software. An observer manually scored scratching and grooming behavior during the first 20 min of the test.

### Home cage observation

Home cage locomotor behavior and stereotypies in a familiar environment were assessed by a 60 min session in the home cage (35 cm L × 15 cm W × 12 cm H) made of transparent plexiglas. For group-housed animals, observations were made for one mouse at a time, with cage mates removed to a temporary cage and 30 min of additional habituation to the home cage once alone. Locomotor behavior was recorded using an overhead camera and analyzed using the ANY-maze (Stoelting Co.) behavior tracking software. Stereotypy behaviors were recorded using an HD video camera positioned level with the cage and at 45° angle to the cage floor, and scored manually during 1-min intervals every 6 min for the total of 10 min of observation. An observer blind to genotype scored video recordings for the number of instances a behavior of interest occurred. We examined grooming (full grooming sequence, from face to hind side), facial only grooming (whisker, ear and face grooming), taffy pulling (repetitive hand to mouth movements), twirling (tight turns, complete circle), circling (follows vaguely circular path, loose turns), route tracing (walks back and forth along the same path), repetitive jumping, repetitive sniffing, rearing (standing up on hind paws, without side of the cage support), gnawing (chewing on things without the purpose of eating them), yawning, swaying (whole body movements from side to side), and sleeping.

### Elevated plus maze test

Exploratory behavior in a novel environment and avoidance behavior were assessed with the elevated plus maze test^81^. The maze consisted of four arms 30 cm long and 5 cm wide, with two open arms and two arms enclosed by 16 cm tall walls painted black. The apparatus was custom made out of Plexiglas plastic painted black and attached to sturdy Plexiglas plastic legs, which elevated it 39 cm off the floor. At the start of the test, the mouse was placed in the center square of the apparatus facing an open arm and allowed to freely explore for 5 min. Behavior was recorded using a video camera positioned directly above the center of the plus maze and analyzed using the ANY-maze (Stoelting Co.) behavior tracking software. An entry into a closed or open arm was counted when the mouse had all four paws in the arm.

### Accelerating rotarod test

The accelerating rotarod test was used to examine motor coordination, balance, and motor learning. Mice were run on a rotarod apparatus (Ugo Basile: 47650) for 4 consecutive days. Three trials were completed per day with a 5 min break between trials. The rotarod was accelerated from 5 to 40 revolutions per minute (r.p.m.) over 300 s for trials 1–6 (days 1 and 2), and from 10 to 80 r.p.m. over 300 s for trials 7–12 (days 3 and 4). On the first testing day, mice were acclimated to the apparatus by being placed on the rotarod rotating at a constant 5 r.p.m. for 60 s and returned to their home cage for 5 min prior to starting trial 1. Latency to fall, or to rotate off the top of the rotarod barrel, was measured by the rotarod stop–trigger timer. Motor coordination was defined as the *y* intercept of the line of best fit from the first to last trial, and motor learning was measured as the slope of performance from the first to last trial for each mouse.

### Three-chamber social approach test

Social approach behavior was assessed using the three-chamber social approach test^82^. The testing apparatus was made of clear Plexiglas (60 cm L × 40 cm W × 22 cm H, Stoelting Co.). Two dividing walls had doorways allowing access to three chambers. Age- and sex-matched C57BL/6J mice were used as novel mice and were habituated to placement inside the wire cups (8 cm diameter, 11 cm tall) prior to testing in either three 10-min or two 15-min sessions. C57BL/6J mice that displayed persistent agitation, including climbing or biting behavior inside the wire cups, were not used for testing.

The test mouse was placed in the center chamber of the apparatus and allowed to explore and habituate to all three empty chambers for 10 min. The mouse was then placed in the center chamber again for 5 min with access doors closed. During this time, a novel C57BL/6J mouse was placed under a wire cup in one of the side chambers and an empty wire cup was placed on the opposite side (novel object). The chamber doors separating the three chambers were then removed to open access to both left and right chambers. The mouse was allowed to explore the three chambers for 10 min, and allowed to interact with the novel object or with the stranger mouse inside the wire cup. The location of the stranger mouse and the novel object were alternated between mice. Behavior was recorded using a video camera positioned directly above the center of the apparatus and analyzed using the ANY-maze (Stoelting Co.) behavior tracking software. An observer blind to genotype manually scored time spent sniffing the novel mouse and the novel object during the test. Sniffing was defined as when the mouse’s nose was perpendicular to the vertical plane and <1 cm away from the wire cup. If the mouse reared on the wire cup, climbed on the wire cup, or bit the wire cup, it was not scored as sniffing. Mice showing a strong side bias during the habituation phase of the test were excluded. Controls were *DAT*^*IRES*^*Cre*-negative littermates of DA-Tsc1-KO mice. Comparisons were made within genotype.

### Rapamycin treatment

Rapamycin (LC Laboratories) was prepared in ethanol at a concentration of 30 mg/ml and stored at −80 °C until used. The stock solution was diluted fresh on the day of injection by first adding 50% volume of 10% PEG-400 with 8% ethanol and then 50% volume of 10% Tween-80. All detergent dilutions were made in sterile saline. Vehicle (the same carrier solution minus rapamycin) or rapamycin were administered intraperitoneally 3 days a week (Monday, Wednesday, and Friday). Rapamycin was dosed at 8 mg/kg and injected starting at 4 weeks of age. Rapamycin was administered for 8–11 weeks prior to perfusion and fixation, including on the day of perfusion.

### Quantification and statistical analysis

Whenever possible, quantification and analyses were performed blind to genotype. All statistical analyses and graphing were performed using the GraphPad Prism 6 or 8 software. All datasets were first analyzed using D’Agostino and Pearson normality test, and then parametric or non-parametric two-tailed statistical tests were employed accordingly to determine significance. If the variances between two groups was significantly different, a Welch’s correction was applied. Significance was set as *p < 0.05, **p < 0.01, ***p < 0.001, and ****p < 0.0001. *P* values were corrected for multiple comparisons. The statistical tests used for each type of experiment are indicated above. Statistical details for each experiment are reported in the figure legends.

### Reporting summary

Further information on research design is available in the Nature Research Reporting Summary linked to this article.

## Supplementary information

Supplementary Information

Reporting Summary

## Data Availability

Source data underlying all figures and tables are provided as a Source Data file.

## Source data

Source Data

## Code availability

Code for the analysis of electron microscopy parameters was generated by Dr. Max Larsson, Linkoping University, and is available at: https://liu.se/medfak/forskning/larsson-max/software?l=en. ScanImage software was generated by Dr. Bernardo Sabatini, Harvard Medical School, and is available at: https://github.com/bernardosabatini/SabalabAcq.
