# Supplementary material for: Tsc1-mTORC1 signaling controls striatal dopamine release and cognitive flexibility
Source: Nat Commun. 2019 Nov 28;10:5426. doi: 10.1038/s41467-019-13396-8 (PMC6882901; doi:10.1038/s41467-019-13396-8)
Supplement: Supplementary file 1 — Supplementary Information [file 41467_2019_13396_MOESM1_ESM.pdf]

Supplementary Information

Tsc1-mTORC1 signaling controls striatal dopamine release and cognitive flexibility

Kosillo et al.

Contents:

Supplementary Figures 1-8

Supplementary Tables 1-10

Supplementary References

Supplementary Figure 1

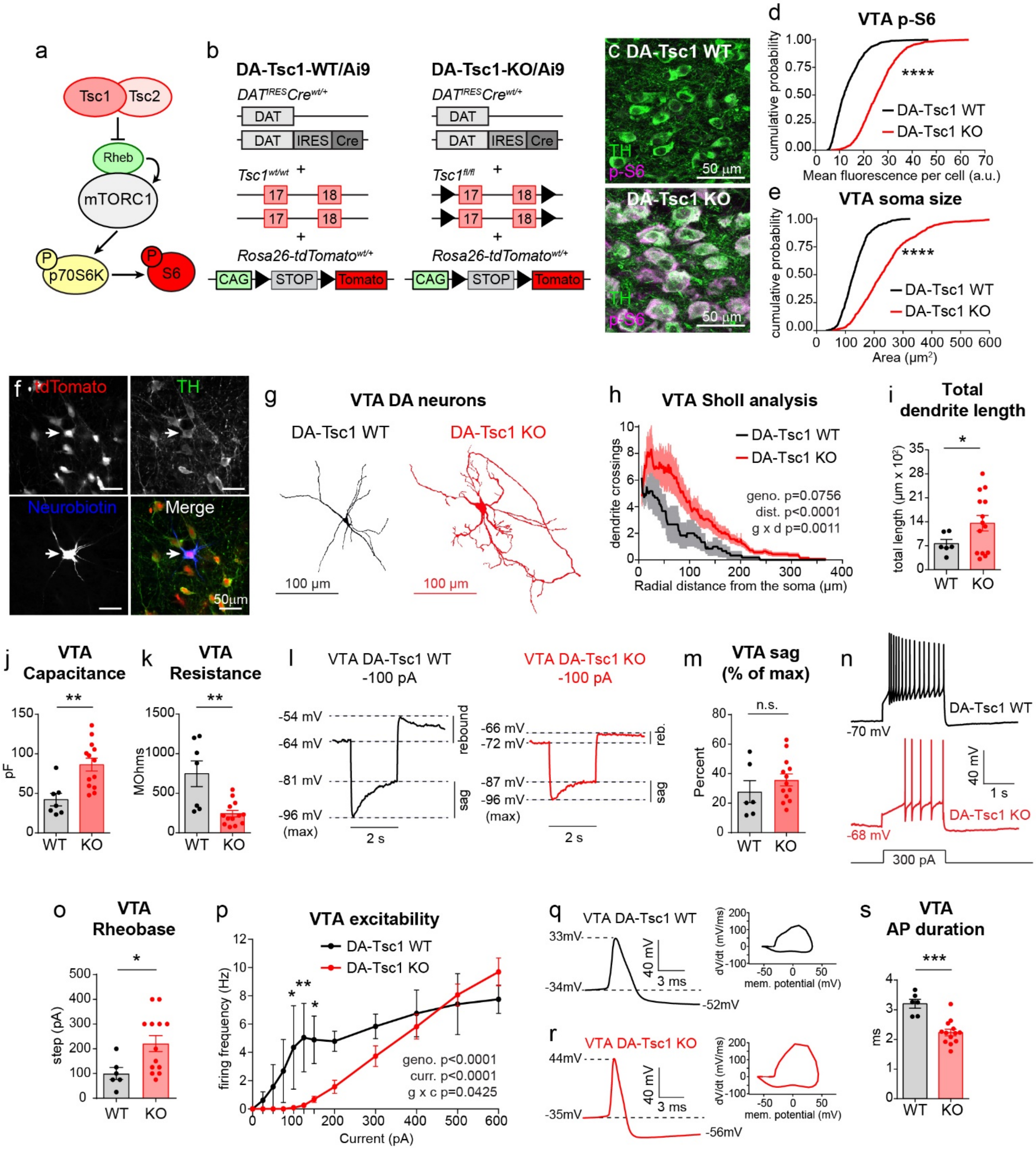

Supplementary Figure 1. DA-Tsc1 KO VTA neurons are hypertrophic and have reduced intrinsic excitability, related to Figures 1 and 2.

(a) Simplified mTORC1 signaling schematic showing the Tsc1/2 heterodimer as a negative regulator of the small GTP-ase Rheb, which directly promotes mTORC1 kinase activity. (b) Schematic of the genetic strategy to selectively delete *Tsc1* from DA neurons, visualized by the Ai9 tdTomato Cre-reporter. (c) Confocal images of VTA DA neurons from DA-Tsc1 WT (top) or DA-Tsc1 KO (bottom) mice. Sections were labelled with antibodies against tyrosine hydroxylase (TH) and phosphorylated S6 (p-S6, Ser240/244). (d,e) Cumulative distribution of VTA DA neuron p-S6 levels (d) and soma area (e) (DA-Tsc1 WT: n=892 neurons from 3 mice, DA-Tsc1 KO: n=863 neurons from 3 mice). ****, p<0.0001, Kolmogorov-Smirnov test. (f) Confocal images of a VTA section containing a triple-labelled (tdTomato Cre-reporter, TH, and neurobiotin) DA neuron. (g) Three-dimensional reconstructions of VTA DA neurons. (h) Sholl analysis of VTA DA neurons. Dark colored lines are the mean, the lighter color shading is SEM (DA-Tsc1 WT: n=6 neurons from 5 mice, DA-Tsc1 KO: n=14 neurons from 8 mice). Two-way ANOVA p values are shown. (i) Mean ± SEM total dendritic length per cell (n is the same as for panel h). *, p=0.0329, Welch's t two-tailed test. (j,k) Mean ± SEM membrane capacitance (j), and membrane resistance (k) (DA-Tsc1 WT: n=6 neurons from 4 mice, DA-Tsc1 KO: n=13 neurons from 8 mice). **, p_{Cm}=0.0024; **, p_{Rm}=0.0047, Mann-Whitney two-tailed tests. (l) Example current-clamp recordings from VTA DA neurons in response to a -100pA current step. (m) Mean ± SEM sag amplitude (DA-Tsc1 WT: n=6 neurons from 4 mice, DA-Tsc1 KO: n=13 neurons from 8 mice). n.s.=not significant, p=0.2441, Mann-Whitney two-tailed test. (n) Examples of action potential firing elicited with a 300 pA current step in VTA DA neurons. (o) Mean ± SEM rheobase of VTA DA neurons calculated as the current at which action potentials were first elicited (DA-Tsc1 WT: n=6 neurons from 4 mice, DA-Tsc1 KO: n=13 neurons from 8 mice). *, p<0.0238, Mann-Whitney two-tailed test. (p) Mean ± SEM excitability curves showing the firing frequency of VTA DA neurons in response to depolarizing current steps of increasing amplitude (DA-Tsc1 WT: n=7 neurons from 4 mice, DA-Tsc1 KO: n=14 neurons from 8 mice). Two-way ANOVA p values are shown. *, p_{100pA}=0.0168; *, p_{150pA}=0.0187; **, p_{125pA}=0.0040; Sidak's multiple comparisons test. (q,r) Examples of individual action potentials and their respective phase plots for VTA DA neurons in DA-Tsc1 WT (q) and DA-Tsc1 KO (r) mice. (s) Mean ± SEM action potential ("AP") duration (DA-Tsc1 WT: n=6 neurons from 4 mice, DA-Tsc1 KO: n=13 neurons from 8 mice). ***, p=0.0003, Mann-Whitney two-tailed test. For all bar graphs, dots indicate values of individual neurons. See also Supplementary Table 2 for complete electrophysiology results. Source data are provided as a Source Data file.

Supplementary Figure 2

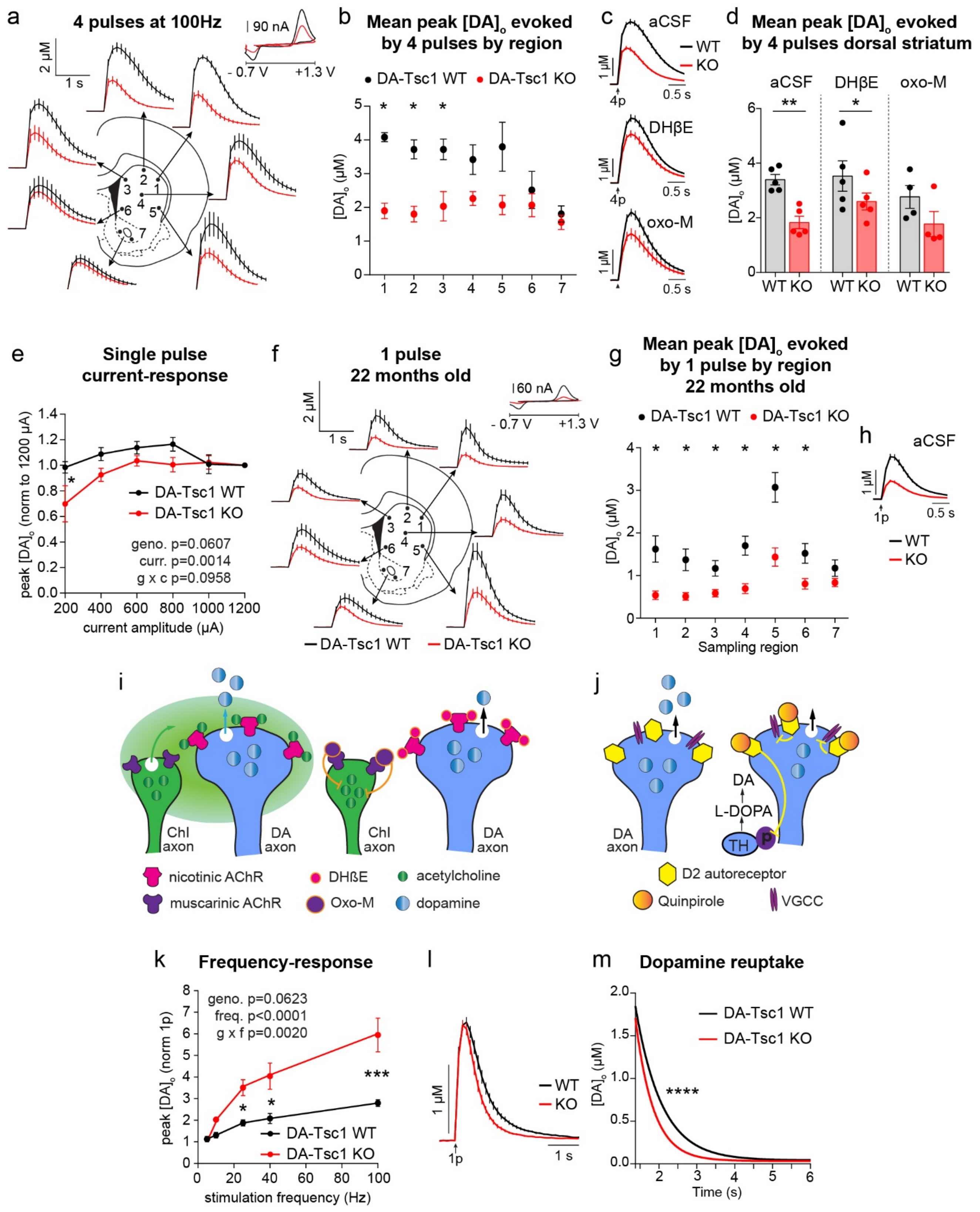

Supplementary Figure 2. *Tsc1* deletion impairs striatal DA release and increases reuptake kinetics, related to Figures 3 and 4.

(a) Mean ± SEM $[DA]_o$ versus time evoked from different striatal subregions by high frequency burst stimulation (4 pulses at 100 Hz). Traces are an average of 7-18 release transients per site from 5 mice per genotype. Inset, typical cyclic voltammograms show characteristic DA waveform. (b) Mean peak ± SEM $[DA]_o$ by striatal region (numbers correspond to the numbered sites in panel a). n=7-18 transients per site from 5 mice per genotype. *, $p_{1,2}$<0.0001; *, p_3=0.0048, paired two-tailed t tests. (c) Mean ± SEM $[DA]_o$ versus time for all dorsal striatum sites recorded in normal aCSF (average of 48-50 transients across 6 recording sites per genotype from 5 mice), 1 μM DHβE (average of 60-61 transients across 6 recording sites per genotype from 5 mice), or 10 μM oxotremorine-M (oxo-M, average of 43-44 transients across 6 recording sites per genotype from 4 mice). (d) Mean peak ± SEM $[DA]_o$ averaged across all dorsal striatum sites (sites #1-6 in panels a and b). Dots represent average per mouse in aCSF (n=5 mice per genotype), 1 μM DHβE (n=5 mice per genotype) and 10 μM oxotremorine-M (n=4 mice per genotype). *, p=0.0334; **, p=0.0022, paired two-tailed t tests. (e) Mean peak ± SEM dorsomedial striatal $[DA]_o$ evoked by single pulse stimulations with current amplitude varying between 200-1200 μA. Data are normalized to 1200 μA within each genotype. n=4 mice per genotype. Two-way ANOVA p values are shown. *, p=0.0140 (for WT vs KO at 200 μA), Sidak's multiple comparisons test. (f) Mean ± SEM $[DA]_o$ versus time evoked from different striatal subregions by single pulse stimulation in aged mice (22 months old). Traces are an average of 16-28 release transients per site from 4 mice per genotype. Inset, typical cyclic voltammograms show characteristic DA waveform. (g) Mean peak ± SEM $[DA]_o$ by striatal region in 22 month old mice (numbers correspond to the numbered sites in panel f). n=16-28 transients per site from 4 mice per genotype. *, p_1=0. 0155; *, p_2=0.0034; *, p_3=0.0335, Wilcoxon two-tailed t tests; *, p_4=0.0014; *, p_5=0.0018, *, p_6=0.0037, paired two-tailed t tests. (h) Mean ± SEM $[DA]_o$ versus time for all dorsal striatum sites recorded in normal aCSF (average of 96 transients across 6 recording sites per genotype from 4 mice) in 22 month old mice. (i) Schematic showing DHβE (nicotinic AChR antagonist) and oxo-M (muscarinic AChR agonist) sites of action at nicotinic receptors on DA axon terminals and muscarinic autoreceptors on cholinergic interneurons, respectively. Both compounds relieve cholinergic control over striatal DA transmission. (j) Schematic of D2 autoreceptor control of striatal DA release via voltage-gated calcium channels (VGCC) and tyrosine hydroxylase (TH) phosphorylation. Quinpirole is a D2 receptor agonist. (k) Mean peak ± SEM dorsolateral striatal $[DA]_o$ evoked by short trains of 4 pulses at 5, 10, 25, 40 and 100 Hz. DHβE (1 μM) applied throughout. Data are normalized to single pulse-evoked $[DA]_o$ within each genotype. n=2 mice per genotype. Two-way ANOVA p values are shown. *, p_{25Hz}=0.0254; *, p_{40Hz}=0.0118; ***, p_{100Hz}=0.0005, Sidak's multiple comparisons test. (l) Mean ± SEM $[DA]_o$ versus time from concentration- and region-matched FCV recordings. Average of 10 transients from 3-4 mice per genotype. (m) Single-phase exponential decay curve-fit of the falling phase of concentration and region-matched DA transients. X-axis starts 375 ms after stimulation onset. n=10 traces from 3-4 mice per genotype. ****, p<0.0001, curve-fit comparison. All data are from 2-4 month old mice, except where noted. Source data are provided as a Source Data file.

Supplementary Figure 3

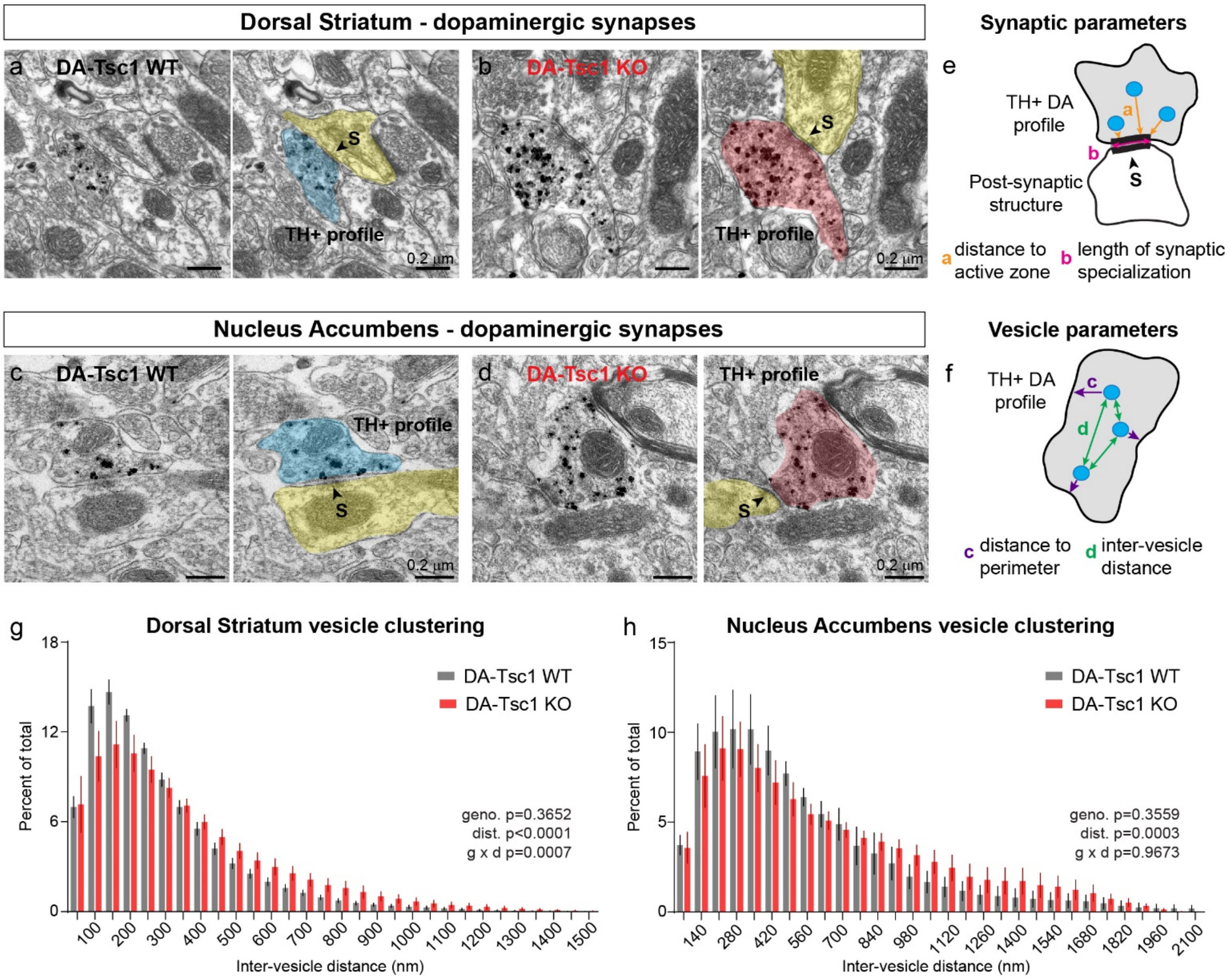

Supplementary Figure 3. Electron micrographs of dopaminergic synaptic profiles, related to Figure 6.

(a-d) Example electron micrographs of dopaminergic axon profiles, identified by enriched immunogold labeling for tyrosine hydroxylase (TH), forming synapses in the dorsolateral striatum (a,b) or nucleus accumbens core (c,d) of DA-Tsc1 WT (a,c) and DA-Tsc1 KO (b,d) mice. Right panels show TH+ axon profiles pseudocolored in blue (DA-Tsc1 WT) or red (DA-Tsc1 KO). Synaptic profiles were identified by the presence of a synaptic membrane specialization (arrowheads, “S”). The post-synaptic spine or dendrite is pseudocolored in yellow. (e) Schematic of vesicle position parameters measured in TH+ profiles with synaptic specializations. (f) Schematic of vesicle position parameters measured within all TH+ axon profiles. (g,h) Histograms of mean ± SEM inter-vesicle distance, averaged per mouse, in dorsolateral striatum binned at 50 nm (g) or NAc core binned at 70 nm (h). Two-way ANOVA p values are shown. See Supplementary Tables 3 and 4 for additional EM analysis. Source data are provided as a Source Data file.

Supplementary Figure 4

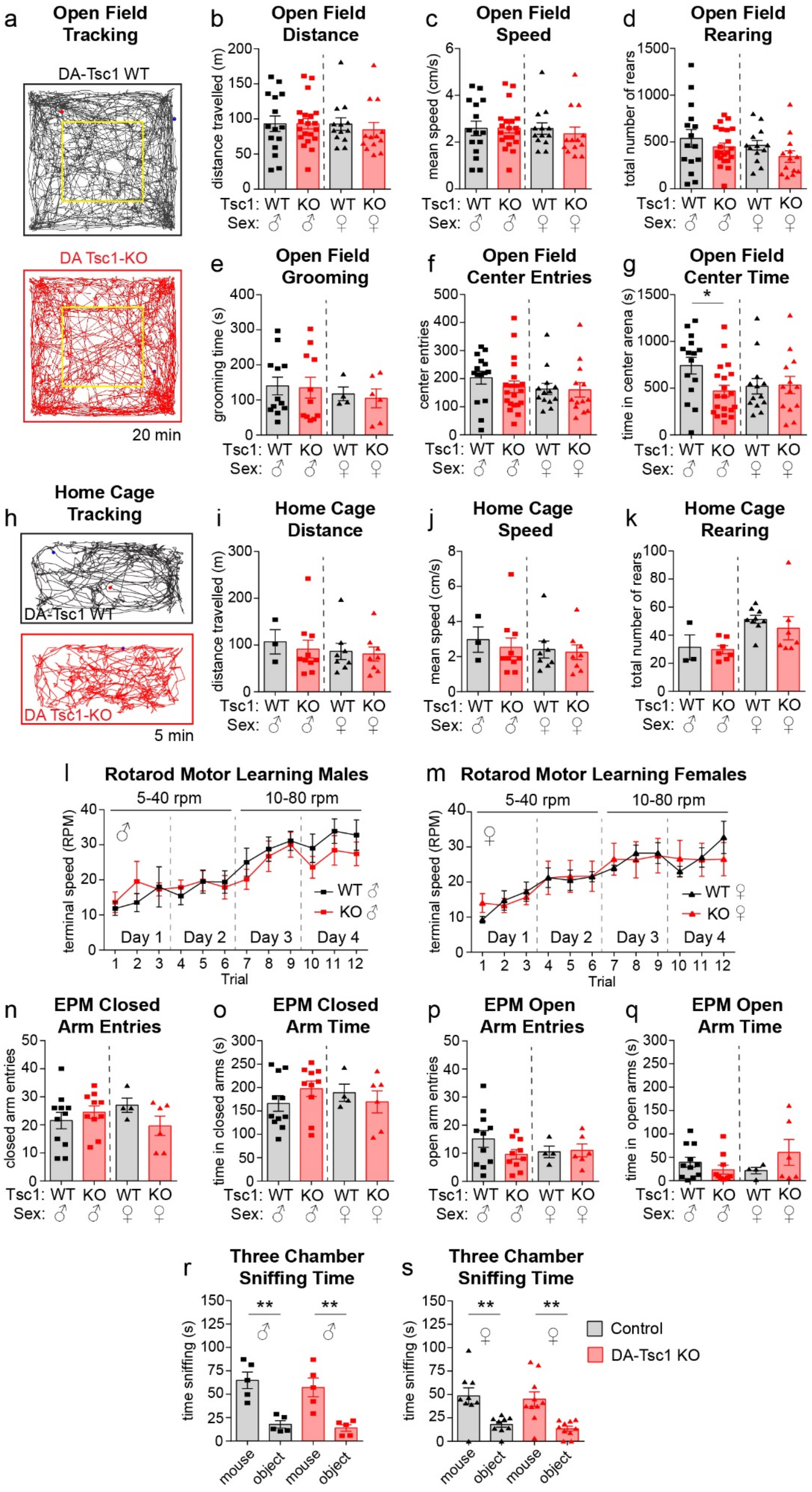

Supplementary Figure 4. DA-Tsc1-KO mice do not show changes in motor behavior or social approach, related to Figure 7.

(a) Examples of automated tracking plots of mouse movement in the open field arena during the first 20 minutes of the test. (b-g) Quantification of open field behavior. Mean ± SEM total distance traveled over 60 minutes (b), mean speed (c), number of rears in 60 minutes (d), time spent grooming during first 20 minutes of the test (e), number of entries into the center zone (outlined in yellow on panel A) over 60 minutes (f), and time spent in the center zone over 60 minutes (g). For panels b-d,f,g, n=15 DA-Tsc1-WT and 20 DA-Tsc1-KO male mice, n=13 DA-Tsc1-WT and 13 DA-Tsc1-KO female mice (except panel d, n=22 DA-Tsc1 KO males). For panel e, n=12 DA-Tsc1-WT and 11 DA-Tsc1-KO male mice, n=4 DA-Tsc1-WT and 6 DA-Tsc1-KO female mice. *, p=0.0142, unpaired, two-tailed t test. (h) Examples of automated tracking plots of mouse movement around the home cage during the first 5 minutes of the test. (i-k) Quantification of home cage behavior. Mean ± SEM total distance traveled over 60 minutes (i), mean speed (j), and number of rears in 10 minutes (k). For panels I & j, n=3 DA-Tsc1-WT and 10 DA-Tsc1-KO male mice, n=8 DA-Tsc1-WT and 8 DA-Tsc1-KO female mice. For panel k, n=3 DA-Tsc1-WT and 7 DA-Tsc1-KO male mice, n=9 DA-Tsc1-WT and 7 DA-Tsc1-KO female mice. Unpaired t tests revealed no significant differences (p>0.05). (l,m) Mean ± SEM terminal speed (speed of the rotarod when the mouse fell off) in revolutions per minute (rpm) for the accelerating rotarod test for male (l) and female (m) mice. n=8 DA-Tsc1 WT and 7 DA-Tsc1 KO male mice, n=4 DA-Tsc1 WT and 6 DA-Tsc1 KO female mice. Two-way ANOVAs revealed no significant effects of genotype (p>0.05). (n-q) Quantification of elevated plus maze (EPM) behavior. Mean ± SEM number of entries into the closed arms (n), time spent in the closed arms (o), entries into the open arms (p), and time spent in the open arms (q) over 5 minutes. n=11 DA-Tsc1 WT and 10 DA-Tsc1 KO male mice, n=4 DA-Tsc1 WT and 6 DA-Tsc1 KO female mice. Unpaired t tests revealed no significant differences (p>0.05). (r,s) Quantification of three chamber social approach test. Mean ± SEM time spent investigating (sniffing) the novel mouse or novel object by male (r) or female (s) mice. Controls are $DAT^{IRES}Cre$ negative littermates of DA-Tsc1 KO mice. Comparisons were made within genotype. n=5 control and 5 DA-Tsc1 KO male mice, n=9 control and 10 DA-Tsc1 KO female mice. **, $p_{Contr\text{-}males}$=0.0069; **, $p_{KO\text{-}males}$=0.0050; **, $p_{Contr\text{-}females}$=0.0047; **, $p_{KO\text{-}females}$=0.0010, paired, two-tailed t tests. For all panels, dots represent values from individual mice. See also Supplementary Tables 5 and 6. Source data are provided as a Source Data file.

Supplementary Figure 5

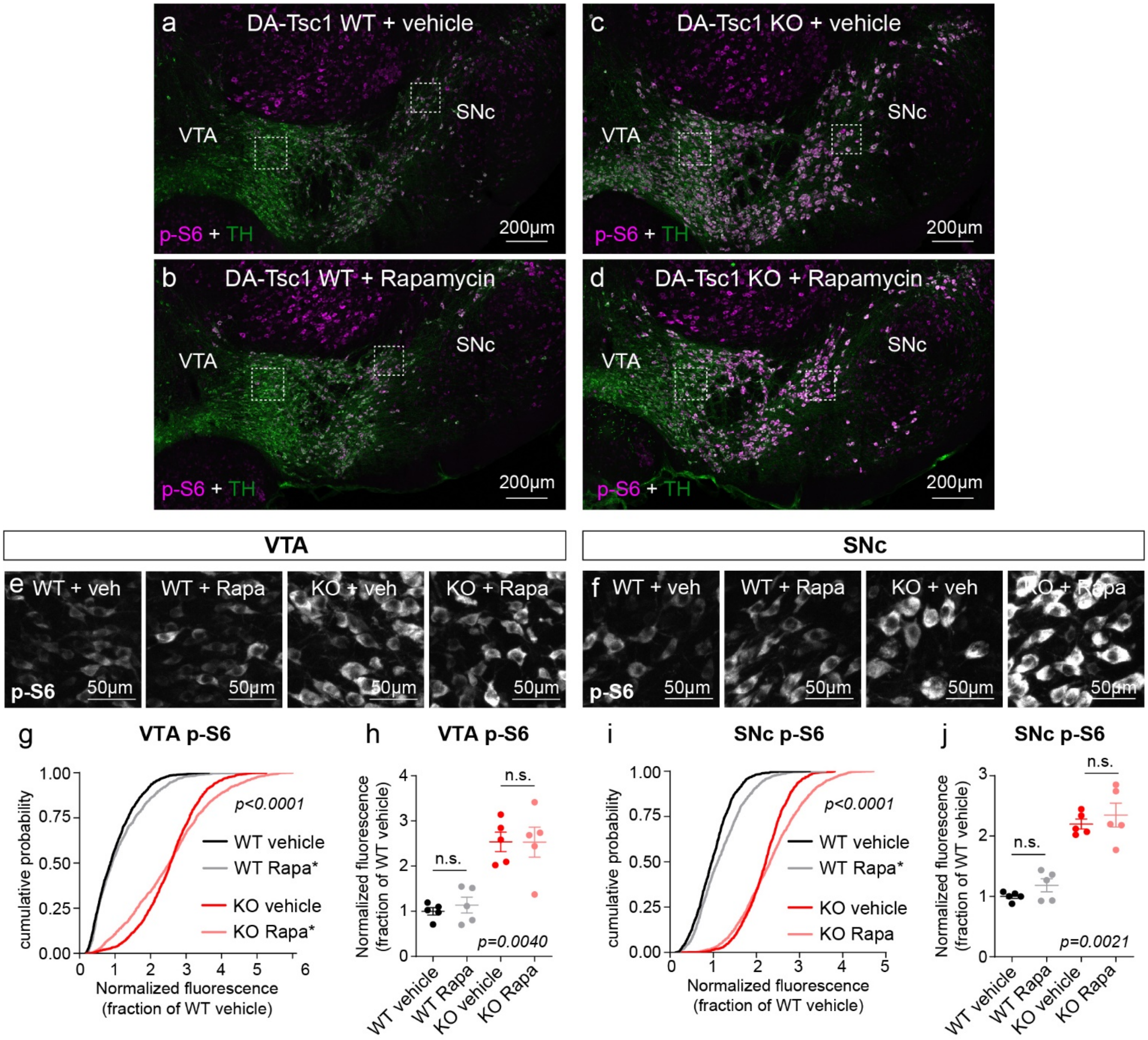

Supplementary Figure 5. Chronic systemic rapamycin treatment does not reduce p-S6 in DA neurons.

(a-d) Confocal images of coronal midbrain sections from DA-Tsc1 WT (a-b) or DA-Tsc1 KO (c-d) mice treated with either vehicle (a,c) or rapamycin (8 mg/kg, 3 times per week, for 8 weeks, b,d). Sections were stained with antibodies against tyrosine hydroxylase (TH, green) and phosphorylated S6 (p-S6, Ser240/244, magenta). (e,f) Panels e and f show higher magnification images of p-S6 immunostaining in greyscale from the boxed regions in a-d. veh=vehicle, Rapa=rapamycin. (g,i) Cumulative distributions of DA neuron p-S6 levels in the VTA (g) and SNc (i) (n=1500 neurons from 5 mice per genotype and treatment). Kruskal-Wallis test p-values are indicated in italics. Dunn's multiple comparisons tests were performed for WT vehicle vs. WT rapamycin ($p=0.0009$ for VTA and $p<0.0001$ for SNc) and KO vehicle vs. KO rapamycin ($p=0.0100$ for VTA and $p=0.1077$ for SNc). Asterisks indicate significant differences between vehicle and rapamycin treatment within genotype. (h,j) The same data from panels g and i, expressed as the average p-S6 level per mouse for VTA (h) and SNc (j) DA neurons. Bars represent mean ± SEM, dots represent individual mice, n=5 mice per genotype and treatment group. Kruskal-Wallis test p-values are indicated in italics. Dunn's multiple comparisons tests were performed for WT vehicle vs. WT rapamycin and KO vehicle vs. KO rapamycin ($p>0.9999$ for all comparisons). For panels g-j, tissue was processed and imaged in two batches. Data were normalized to the WT vehicle mice within each batch. Source data are provided as a Source Data file.

Supplementary Figure 6

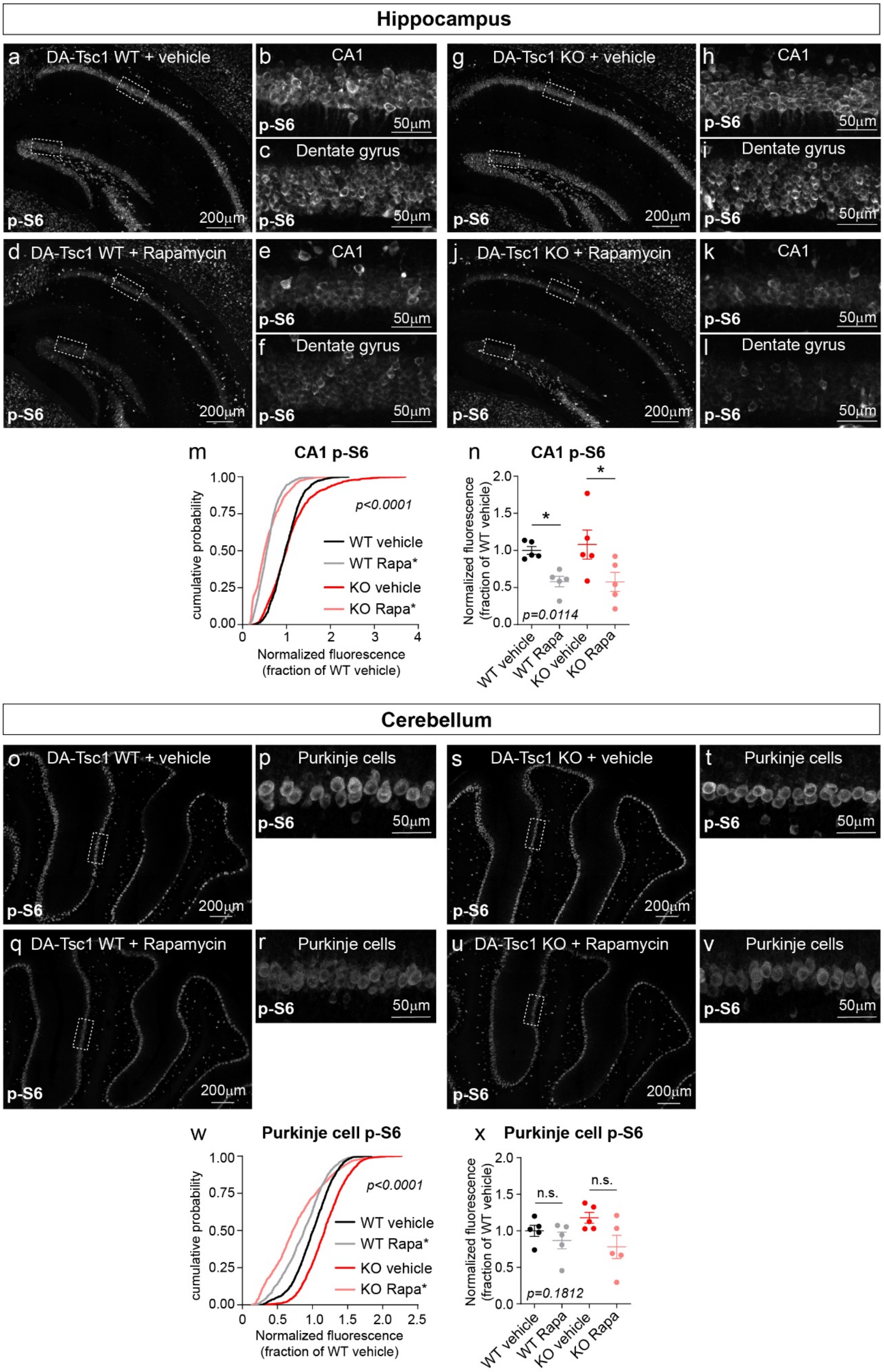

Supplementary Figure 6. Chronic systemic rapamycin reduces p-S6 levels in the hippocampus and cerebellum.

(a-l) Confocal images of hippocampal sections from DA-Tsc1 WT (a-f) or DA-Tsc1 KO (g-l) mice treated with either vehicle (a-c & g-i) or rapamycin (8 mg/kg, 3 times per week, for 8 weeks, d-f & j-l). Sections were stained with an antibody against phosphorylated S6 (p-S6, Ser240/244). Panels to the right show higher magnification images of the boxed regions. (m) Cumulative distributions of CA1 neuron p-S6 levels (n=1500 neurons from 5 mice per genotype and treatment). Kruskal-Wallis test p-value is indicated in italics. Multiple comparisons tests were performed for WT vehicle vs. WT rapamycin ($p<0.0001$) and KO vehicle vs. KO rapamycin ($p<0.0001$). Asterisks indicate significant differences between vehicle and rapamycin treatment within genotype. (n) The same data as in panel m expressed as the average p-S6 level per mouse. Bars represent mean ± SEM, dots represent individual mice, n=5 mice per genotype and treatment group. Kruskal-Wallis test p-value is indicated in italics. Dunn's multiple comparisons tests were performed for WT vehicle vs. WT rapamycin ($p=0.0431$) and KO vehicle vs. KO rapamycin ($p=0.0323$). (o-v) Confocal images of cerebellar sections from mice of the indicated genotype and treatment, stained with an antibody against p-S6. (w) Cumulative distributions of Purkinje cell p-S6 levels (n=1500 neurons from 5 mice per genotype and treatment). Kruskal-Wallis test p-value is indicated in italics. Dunn's multiple comparisons tests were performed for WT vehicle vs. WT rapamycin ($p<0.0001$) and KO vehicle vs. KO rapamycin ($p<0.0001$). (x) The same data as in panel m, expressed as the average p-S6 level per mouse. Bars represent mean ± SEM, dots represent individual mice, n=5 mice per genotype and treatment group. Kruskal-Wallis test p-value is indicated in italics. Dunn's multiple comparisons tests were performed for WT vehicle vs. WT rapamycin ($p>0.9999$) and KO vehicle vs. KO rapamycin ($p=0.0845$). Source data are provided as a Source Data file.

Supplementary Figure 7

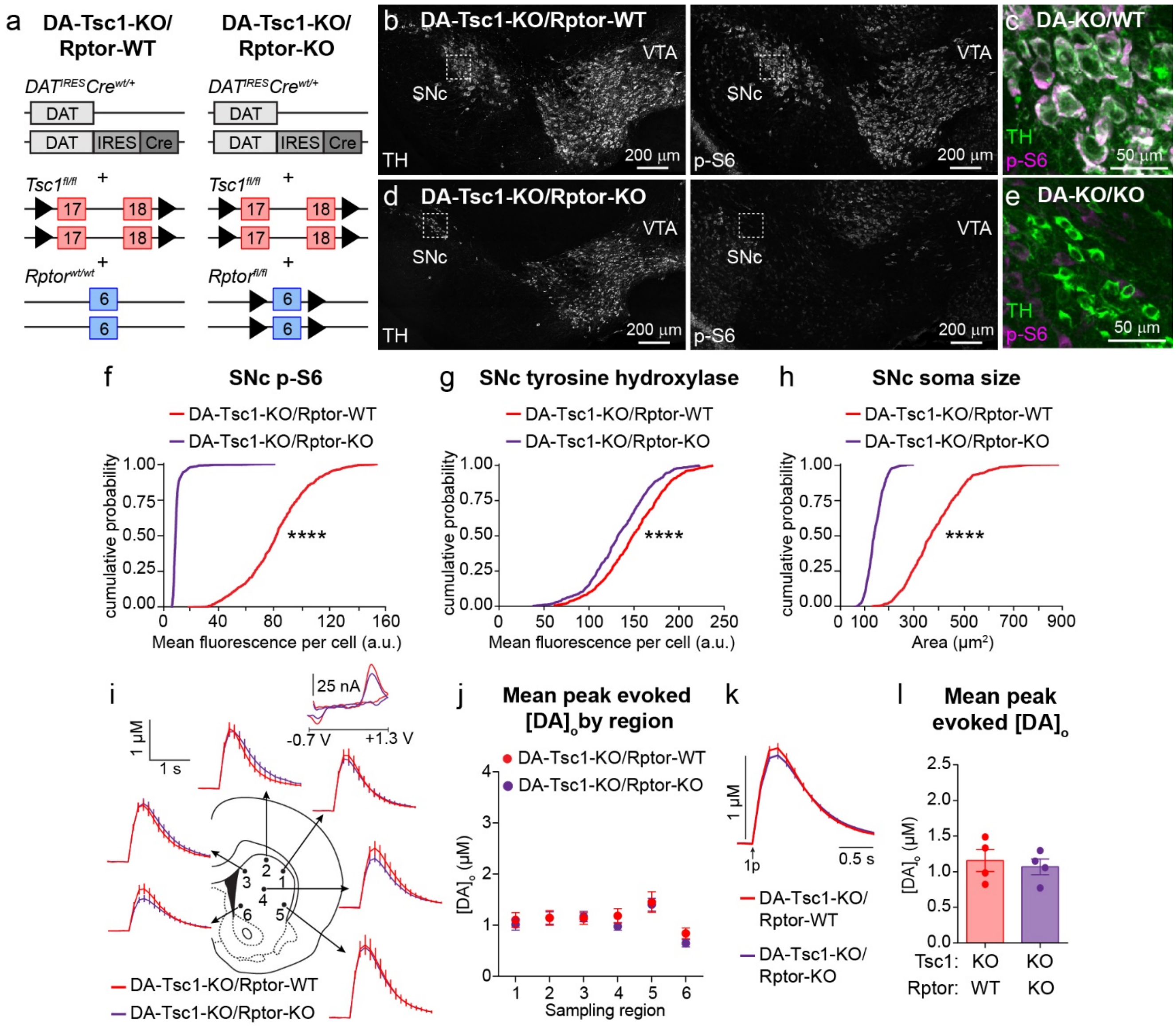

Supplementary Figure 7. Homozygous deletion of *Rptor* from DA-Tsc1 KO neurons causes somatic hypotrophy and does not prevent dopamine release deficits, related to Figure 8.

(a) Schematic of the genetic strategy to selectively delete *Tsc1* and *Rptor* in DA neurons. (b-e) Confocal images of coronal midbrain sections from DA-Tsc1-KO/Rptor-WT (b,c) and DA-Tsc1-KO/Rptor-KO (d,e) mice. Sections were labelled with antibodies against tyrosine hydroxylase (TH) and phosphorylated S6 (p-S6, Ser240/244). Panels c and e show higher magnification merged images of the boxed regions in b and d. (f-h) Cumulative distributions of SNc DA neuron p-S6 levels (f), tyrosine hydroxylase (TH) levels (g), and soma area (h) (DA-Tsc1-KO/Rptor-WT: n=530 neurons from 3 mice, DA-Tsc1-KO/Rptor-KO: n=669 neurons from 3 mice). ****, $p<0.0001$, Kolmogorov-Smirnov tests. (i) Mean ± SEM $[DA]_o$ versus time evoked from different dorsal striatal subregions by single-pulse electrical stimuli. Traces show an average of 16 transients per recording site from 4 mice per genotype. Inset, typical cyclic voltammograms show characteristic DA waveform. (j) Mean ± SEM peak $[DA]_o$ by striatal region (numbers correspond to the numbered sites in panel i). n=16 transients per recording site from 4 mice per genotype. Paired two-tailed t tests revealed no significant differences ($p>0.05$). (k) Mean ± SEM $[DA]_o$ versus time for all dorsal striatum sites (sites #1-6 in panels i and j). Average of 96 transients across 6 recording sites per genotype from 4 mice. (l) Mean ± SEM peak $[DA]_o$ averaged across all dorsal striatum sites (sites #1-6 in panels i and j). Dots represent average peak evoked $[DA]_o$ per mouse. n=4 mice per genotype. Paired two-tailed t test revealed no significant difference ($p>0.05$). Source data are provided as a Source Data file.

Supplementary Figure 8

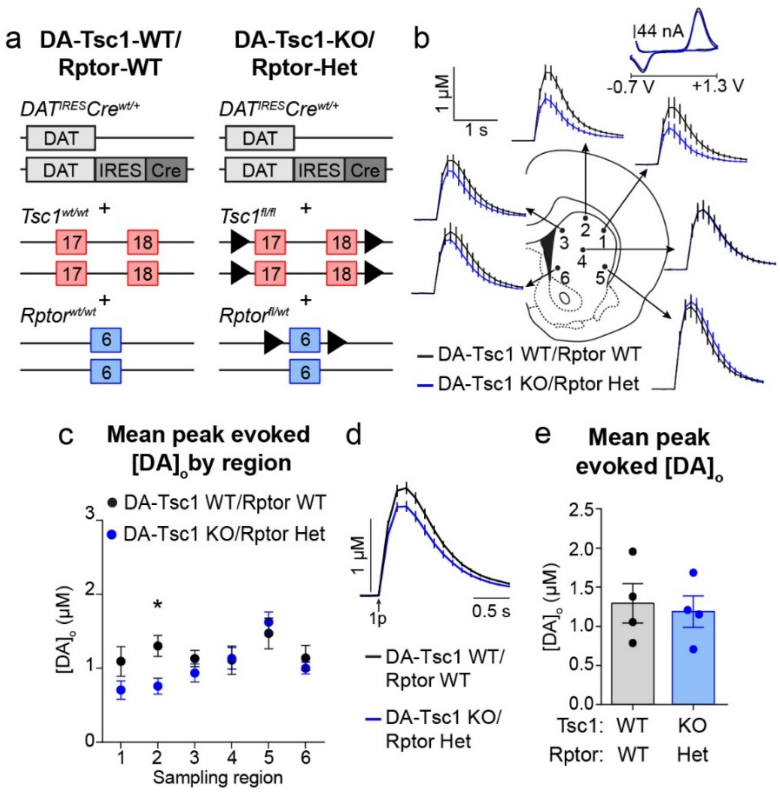

Supplementary Figure 8. Heterozygous deletion of *Rptor* from DA-Tsc1 KO neurons normalizes evoked dopamine release to control levels, related to Figure 8.

(a) Schematic of the genetic strategy to selectively delete *Tsc1* and reduce *Rptor* by one copy in DA neurons. (b) Mean ± SEM $[DA]_o$ versus time evoked from different dorsal striatal subregions by single-pulse electrical stimuli. Traces show an average of 16 transients per recording site from 4 mice per genotype. Inset, typical cyclic voltammograms show characteristic DA waveform. (c) Mean ± SEM peak $[DA]_o$ by striatal region (numbers correspond to the numbered sites in panel b). n=16 transients per recording site from 4 mice per genotype. *, p=0.0135, paired two-tailed t test. (d) Mean ± SEM $[DA]_o$ versus time for all dorsal striatum sites (sites #1-6 in panels b and c). Average of 96 transients across 6 recording sites per genotype from 4 mice. (e) Mean ± SEM peak $[DA]_o$ averaged across all dorsal striatum sites (sites #1-6 in panels b and c). Dots represent average peak evoked $[DA]_o$ per mouse. n=4 mice per genotype. Paired t test revealed no significant difference (p>0.05). Source data are provided as a Source Data file.

Supplementary Table 1. Summary of electrophysiology data for SNc DA neurons, related to Figures 1 and 2 (source data are provided as a Source Data file)

	DA-Tsc1 WT (SNc)				DA-Tsc1 KO (SNc)				WT vs KO
Properties	Mean	SEM	n (cells)	n (mice)	Mean	SEM	n (cells)	n (mice)	p-value/ test
**Holding current** at -70mV (pA)	-203.06	28.36	18	5	-313.23	20.04	31	9	**0.0022** Unpaired t-test
**Series resistance** (mOhms)	9.51	1.42	18	5	8.93	0.72	31	9	0.6293 Mann-Whitney
**Membrane resistance** (mOhms)	222.83	30.90	18	5	101.97	8.09	31	9	**< 0.0001** Mann-Whitney
**Membrane capacitance** (pF)	64.35	3.82	18	5	128.67	6.28	31	9	**< 0.0001** Welch's t-test
**Resting membrane potential** (mV)	-56.13	1.13	18	5	-53.62	2.01	29	9	0.6301 Unpaired t-test
**Rheobase –** (current when first action potentials occur, pA)	145.83	18.66	18	5	341.96	24.70	28	9	**< 0.0001** Welch's t-test
**Action potential threshold** (mV)	-28.70	1.40	18	5	-32.37	1.00	28	9	**0.0345** Unpaired t-test
**Action potential width at threshold** (ms)	2.77	0.11	18	5	1.94	0.03	28	9	**< 0.0001** Welch's t-test
**Action potential peak** (maximum membrane potential, mV)	33.14	1.12	18	5	33.80	0.95	28	9	0.5546 Mann-Whitney
**Action potential height** (change in membrane potential from the start of the spike to maximum depolarization, mV)	73.86	1.07	18	5	75.14	0.94	28	9	0.3200 Mann-Whitney
**Afterhyperpolarization** (minimum membrane potential after the spike, mV)	-59.08	1.38	18	5	-60.89	0.85	28	9	0.2415 Unpaired t-test
**Afterhyperpolarization** (change in membrane potential from the start of the spike to maximum hyperpolarization, mV)	18.36	0.91	18	5	19.56	0.67	28	9	0.2851 Unpaired t-test
**Maximum hyperpolarization in response to -100 pA**	-91.51	2.58	18	5	-79.42	0.96	31	9	**0.0002** Welch's t-test

(from ~-70 mV in response to a 2 second -100 pA current step, mV)									
**Sag component in response to -100 pA** (maximum hyperpolarization minus the steady state membrane potential in the last 50 ms of the current step, mV)	9.82	0.98	18	5	5.50	0.45	31	9	**0.0002** Mann-Whitney
**Sag component expressed as a percentage** (sag component as a percentage of the total step size, calculated as the difference between the max hyperpolarization and baseline potential, %)	45.90	2.82	18	5	52.51	2.45	31	9	0.0941 Unpaired t-test
**Rebound depolarization in response to -100 pA** (baseline membrane potential minus the maximum depolarization within 500 ms of the end of the current step, mV)	7.10	0.61	18	5	4.24	0.24	31	9	**0.0002** Welch's t-test
**Rebound depolarization expressed as a percentage** (rebound as a percentage of the total step size, calculated as the difference between the max hyperpolarization and baseline potential, %)	34.62	2.56	18	5	42.63	2.36	31	9	**0.0341** Unpaired t-test

Supplementary Table 2. Summary of electrophysiology data for VTA DA neurons, related to Supplementary Figure 1 (source data are provided as a Source Data file)

	**DA-Tsc1 WT (VTA)**				**DA-Tsc1 KO (VTA)**				**WT vs KO**
Properties	Mean	SEM	n (cells)	n (mice)	Mean	SEM	n (cells)	n (mice)	p-value/ test
**Holding current** at -70mV (pA)	-117.14	29.34	7	4	-189.23	35.40	13	8	**0.0186** Mann-Whitney
**Series resistance** (mOhms)	7.93	1.69	7	4	8.48	0.78	13	8	0.3507 Mann-Whitney
**Membrane resistance** (mOhms)	746.91	162.31	7	4	241.24	41.31	13	8	**0.0047** Mann-Whitney
**Membrane capacitance** (pF)	42.14	7.90	7	4	86.32	8.10	13	8	**0.0024** Mann-Whitney
**Resting membrane potential** (mV)	-55.64	2.19	7	4	-57.99	1.40	12	8	0.5918 Mann-Whitney
**Rheobase –** (current when first action potentials occur, pA)	89.29	23.05	7	4	223.08	31.71	13	8	**0.0238** Mann-Whitney
**Action potential threshold** (mV)	-32.24	1.80	6	4	-32.15	1.14	13	8	0.8314 Mann-Whitney
**Action potential width at threshold** (ms)	3.20	0.15	6	4	2.23	0.11	13	8	**0.0003** Mann-Whitney
**Action potential peak** (maximum membrane potential, mV)	33.82	1.90	6	4	38.87	1.31	13	8	**0.0365** Mann-Whitney
**Action potential height** (change in membrane potential from the start of the spike to maximum depolarization, mV)	75.24	1.88	6	4	78.78	1.23	13	8	0.1061 Mann-Whitney
**Afterhyperpolarization** (minimum membrane potential after the spike, mV)	-56.99	2.00	6	4	-61.32	1.42	13	8	0.1274 Mann-Whitney
**Afterhyperpolarization** (change in membrane potential from the start of the spike to maximum hyperpolarization, mV)	15.57	1.90	6	4	21.42	0.98	13	8	**0.0285** Mann-Whitney
**Maximum hyperpolarization in response to -100 pA** (from ~-70 mV in response to a 2 second	-112.2	8.51	6	4	-91.98	3.28	13	8	**0.0462** Mann-Whitney

-100 pA current step, mV)									
**Sag component in response to -100 pA** (maximum hyperpolarization minus the steady state membrane potential in the last 50 ms of the current step, mV)	11.29	2.58	6	4	7.48	1.16	13	8	0.2441 Mann-Whitney
**Sag component expressed as a percentage** (sag component as a percentage of the total step size, calculated as the difference between the max hyperpolarization and baseline potential, %)	27.81	7.47	6	4	35.71	4.08	13	8	0.2441 Mann-Whitney
**Rebound depolarization in response to -100 pA** (baseline membrane potential minus the maximum depolarization within 500 ms of the end of the current step, mV)	5.18	1.51	6	4	4.69	0.62	13	8	0.7654 Mann-Whitney
**Rebound depolarization expressed as a percentage** (rebound as a percentage of the total step size, calculated as the difference between the max hyperpolarization and baseline potential, %)	13.30	4.50	6	4	25.40	4.29	13	8	0.0874 Mann-Whitney

Supplementary Table 3. Summary of dorsal striatum electron microscopy analysis, related to Figure 6 (source data are provided as a Source Data file)

	**DA-Tsc1 WT**	**DA-Tsc1 KO**	**WT vs KO**
	Mean ± SEM	Mean ± SEM	p-value/ test
**All profiles** (n=239 profiles for DA-Tsc1 WT and 252 for DA-Tsc1 KO, from 4 mice per genotype)			
Perimeter (µm)	1.73 ± 0.05	2.56 ± 0.08	**< 0.0001** K-S test
Area (µm²)	0.127 ± 0.005	0.296 ± 0.015	**< 0.0001** K-S test
Number of vesicles (total per profile)	126.29 ± 4.10	92.86 ± 3.73	**< 0.0001** K-S test
Vesicle density (per µm²)	1217.64 ± 39.40	577.64 ± 35.93	**< 0.0001** K-S test
Vesicle distance to perimeter (average distance per profile, nm)	43.56 ± 0.83	65.89 ± 1.69	**< 0.0001** K-S test
Inter-vesicle distance (average per profile, nm)	210.50 ± 5.95	290.2 ± 8.47	**< 0.0001** K-S test
Synaptic incidence (average per mouse, %)	18.86 ± 4.18	15.19 ± 5.31	> 0.9999 Mann-Whitney
**Profiles with synaptic specializations** (n=45 profiles from 4 mice for DA-Tsc1 WT and 39 profiles from 3 mice for DA-Tsc1 KO)			
Perimeter (µm)	1.69 ± 0.10	2.06 ± 0.13	0.1704 K-S test
Area (µm²)	0.119 ± 0.010	0.213 ± 0.025	**0.0151** K-S test
Length of synaptic membrane specialization (nm)	63.51 ± 3.98	71.94 ± 6.26	0.4394 K-S test
Number of vesicles (total per profile)	158.70 ± 11.60	185.6 ± 11.62	0.1147 K-S test
Vesicle density (per µm²)	1616.90 ± 122.20	1204.12 ± 105.14	0.0664 K-S test
Vesicle distance to perimeter (average distance per profile, nm)	41.34 ± 1.81	53.58 ± 3.16	**0.0044** K-S test
Inter-vesicle distance (average per profile, nm)	217.3 ± 13.23	267.1 ± 16.72	0.0949 K-S test
Vesicle distance to the active zone (average per profile, nm)	200.7 ± 13.63	252.3 ± 17.49	0.1191 K-S test
Number of vesicles <25nm from the active zone (total per profile)	5.00 ± 0.52	4.95 ± 0.53	0.9639 K-S test

Supplementary Table 4. Summary of nucleus accumbens electron microscopy analysis, related to Figure 6 (source data are provided as a Source Data file)

	DA-Tsc1 WT	DA-Tsc1 KO	WT vs KO
	Mean ± SEM	Mean ± SEM	p-value/ test
**All profiles** (n=120 profiles for DA-Tsc1 WT and 120 for DA-Tsc1 KO, from 4 mice per genotype)			
Perimeter (μm)	3.09 ± 0.13	3.34 ± 0.11	0.0507 K-S test
Area (μm²)	0.453 ± 0.034	0.516 ± 0.028	**0.0011** K-S test
Number of vesicles (total per profile)	55.01 ± 2.80	58.98 ± 2.33	**0.0354** K-S test
Vesicle density (per μm²)	151.70 ± 6.81	135.90 ± 6.03	0.0987 K-S test
Vesicle distance to perimeter (average distance per profile, nm)	78.45 ± 2.10	87.93 ± 2.22	**0.0011** K-S test
Inter-vesicle distance (average per profile, nm)	419.9 ± 19.54	446.40 ± 15.39	**0.0072** K-S test
Synaptic incidence (average per mouse, %)	12.50 ± 2.17	15.83 ± 1.82	0.4857 Mann-Whitney
**Profiles with synaptic specializations** (n=15 profiles for DA-Tsc1 WT and 19 for DA-Tsc1 KO, from 4 mice per genotype)			
Perimeter (μm)	3.25 ± 0.29	3.54 ± 0.27	0.6242 K-S test
Area (μm²)	0.531 ± 0.060	0.671 ± 0.111	0.6072 K-S test
Length of synaptic membrane specialization (nm)	185.2 ± 24.80	159.1 ± 17.51	0.6072 K-S test
Number of vesicles (total per profile)	69.60 ± 10.37	66.89 ± 5.59	0.5074 K-S test
Vesicle density (per μm²)	139.46 ± 14.87	129.33 ± 16.72	0.8372 K-S test
Vesicle distance to perimeter (average distance per profile, nm)	89.25 ± 4.68	92.63 ± 7.79	>0.9999 K-S test
Inter-vesicle distance (average per profile, nm)	447.8 ± 45.93	495.3 ± 43.49	0.5566 K-S test
Vesicle distance to the active zone (average per profile, nm)	715.16 ± 71.72	764.13 ± 81.42	0.9242 K-S test
Number of vesicles <25nm from the active zone (total per profile)	1.53 ± 0.41	1.21 ± 0.25	0.9933 K-S test

Supplementary Table 5. Summary of behavior testing results, related to Figures 7, 9, and Supplementary Figure 4 (source data are provided as a Source Data file)

	DA-Tsc1 WT	DA-Tsc1 KO	WT vs KO
	Mean ± SEM	Mean ± SEM	p-value/ test
**Open Field Test** (60 minutes) (WT n=28, KO n=33 mice; except rears KO n=35 mice)			
Distance travelled (m)	92.94 ± 7.12	89.83 ± 6.03	0.7383 Unpaired t-test
Travelling speed (cm/s)	2.58 ± 0.20	2.50 ± 0.17	0.7504 Unpaired t-test
Number of rears	503.80 ± 55.74	408.80 ± 35.23	0.1400 Unpaired t-test
Time spent in the center (s)	639.00 ± 63.81	494.30 ± 51.59	0.0799 Unpaired t-test
Number of center entries	184.90 ± 15.59	166.70 ± 16.05	0.1904 Mann-Whitney
**Open Field Test** (20 minutes) (WT n=16, KO n=17 mice)			
Time spent grooming (s)	134.10 ± 19.30	124.1 ± 21.19	0.7291 Unpaired t-test
**Home Cage Observation** (60 minutes) (WT n=11, KO n=18 mice)			
Distance travelled (m)	91.91 ± 14.08	86.54 ± 12.23	0.7354 Mann-Whitney
Travelling speed (cm/s)	2.56 ± 0.39	2.41 ± 0.34	0.6821 Mann-Whitney
**Home Cage Observation** (10 minutes) (WT n=12, KO n=14 mice)			
Number of rears	46.33 ± 3.85	37.36 ± 4.72	**0.0477** Mann-Whitney
Number of grooming bouts	5.83 ± 1.48	7.64 ± 1.78	0.3250 Mann-Whitney
Number of facial grooming bouts	6.33 ± 1.10	7.43 ± 1.00	0.4667 Unpaired t-test
Number of taffy pulling occurrences	3.42 ± 0.87	2.14 ± 0.54	0.2143 Unpaired t-test
Number of circling occurrences	1.17 ± 0.57	1.07 ± 0.34	0.7584 Mann-Whitney
Number of route tracing occurrences	1.08 ± 0.36	0.43 ± 0.29	0.1217 Mann-Whitney
Number of jumping occurrences	12.25 ± 4.36	6.78 ± 4.57	0.1660 Mann-Whitney
Number of sniffing occurrences	61.92 ± 3.98	52.64 ± 4.70	0.1085 Mann-Whitney
Number of gnawing occurrences	0.92 ± 0.38	1.29 ± 0.46	0.5511 Unpaired t-test
**Elevated Plus Maze** (5 minutes) (WT n=15, KO n=16 mice)			
Closed arm entries	23.00 ± 2.30	22.69 ± 1.95	0.9177 Unpaired t-test

Open arm entries	13.87 ± 2.27	10.13 ± 1.32	0.1595<br>Unpaired t-test
Closed arm time (s)	172.20 ± 13.14	187.10 ± 13.65	0.4399<br>Unpaired t-test
Open arm time (s)	34.69 ± 8.12	37.47 ± 12.27	0.7035<br>Mann-Whitney
	**Novel object**	**Novel mouse**	**Object vs. mouse (within genotype)**
	Mean ± SEM	Mean ± SEM	p-value/<br>test
**Three Chamber Test** (10 minutes) (Control n=14, KO n=15 mice)			
Time in chamber (s) – Control	180.90 ± 19.62	309.50 ± 33.61	**0.0192**<br>Paired t-test
Time in chamber (s) – DA-Tsc1 KO	151.80 ± 24.30	303.9 ± 28.30	**0.0017**<br>Wilcoxon
Time spent sniffing (s) – Control	17.93 ± 2.24	54.39 ± 6.55	**<0.0001**<br>Paired t-test
Time spent sniffing (s) – DA-Tsc1 KO	13.66 ± 2.06	49.18 ± 6.16	**<0.0001**<br>Paired t-test
	**DA-Tsc1 WT**	**DA-Tsc1 KO**	**WT vs KO**
	Mean ± SEM	Mean ± SEM	adjusted p-value (Sidak's multiple comparisons test)
**Rotarod Test** (WT n=12, KO n=13 mice)			
Terminal speed trial 1 (rpm)	10.80 ± 1.25	13.82 ± 1.90	0.9989
Terminal speed trial 2 (rpm)	14.13 ± 1.78	15.80 ± 2.62	0.9999
Terminal speed trial 3 (rpm)	17.66 ± 3.23	16.48 ± 1.38	0.9999
Terminal speed trial 4 (rpm)	17.33 ± 2.00	19.25 ± 2.26	0.9999
Terminal speed trial 5 (rpm)	19.83 ± 2.33	20.50 ± 2.44	0.9999
Terminal speed trial 6 (rpm)	20.15 ± 2.06	19.64 ± 2.63	0.9999
Terminal speed trial 7 (rpm)	24.67 ± 2.63	23.08 ± 2.66	0.9999
Terminal speed trial 8 (rpm)	28.58 ± 2.37	26.54 ± 3.21	0.9999
Terminal speed trial 9 (rpm)	30.17 ± 2.04	28.85 ± 2.88	0.9999
Terminal speed trial 10 (rpm)	27.00 ± 2.80	25.00 ± 2.84	0.9999
Terminal speed trial 11 (rpm)	31.58 ± 2.63	27.46 ± 3.02	0.9640
Terminal speed trial 12 (rpm)	32.75 ± 3.14	27.00 ± 2.73	0.7287
	**DA-Tsc1 WT**	**DA-Tsc1 KO**	**WT vs KO**
	Mean ± SEM	Mean ± SEM	p-value/<br>test
**Four Choice Reversal Task** (WT n=8, KO n=10 mice; males only)			
Trials to criterion - acquisition	20.25 ± 2.66	24.20 ± 1.88	0.2315<br>Unpaired t-test
Total errors during acquisition	8.12 ± 1.64	9.20 ± 1.04	0.5732<br>Unpaired t-test

Trials to criterion - reversal	34.50 ± 3.19	46.20 ± 3.89	**0.0392** Unpaired t-test
Total errors during reversal	20.50 ± 2.47	32.50 ± 3.77	**0.0230** Unpaired t-test
Rewards consumed in acquisition	12.25 ± 1.18	15.00 ± 1.12	0.1115 Unpaired t-test
Rewards consumed in reversal	14.00 ± 1.31	13.70 ± 0.72	0.8346 Unpaired t-test
Trials to criterion – after first choice of odor 2 (clove)	24.13 ± 2.99	24.60 ± 2.01	0.8935 Unpaired t-test
	**DA-Tsc1 WT**	**DA-Tsc1 KO**	**WT vs KO**
	Mean ± SEM	Mean ± SEM	adjusted p-value (Sidak's multiple comparisons test)
Perseverative errors	10.13 ± 1.87	20.10 ± 3.32	**<0.0001**
Regressive errors	7.13 ± 1.47	7.20 ± 1.25	0.9999
Novel errors	1.88 ± 0.48	1.40 ± 0.50	0.9998
Irrelevant errors	1.25 ± 0.41	2.00 ± 0.42	0.9983
Omissions	0.13 ± 0.13	1.80 ± 0.79	0.9381
	**DA-Tsc1 KO/ Rptor WT**	**DA-Tsc1 KO/ Rptor Het**	**KO/WT vs KO/Het**
	Mean ± SEM	Mean ± SEM	p-value/ test
**Four Choice Reversal Task** (KO/WT n=8, KO/HET n=11 mice; males only)			
Trials to criterion - acquisition	32.13 ± 5.87	30.00 ± 3.96	0.7589 Unpaired t-test
Total errors during acquisition	16.00 ± 3.95	15.00 ± 2.46	0.8238 Unpaired t-test
Trials to criterion - reversal	46.88 ± 4.53	33.18 ± 3.84	**0.0338** Unpaired t-test
Total errors during reversal	31.50 ± 3.97	18.73 ± 2.38	**0.0095** Unpaired t-test
Rewards consumed in acquisition	16.38 ± 1.94	15.00 ± 1.81	0.6126 Mann-Whitney
Rewards consumed in reversal	15.38 ± 1.95	14.45 ± 1.87	0.7422 Unpaired t-test
Trials to criterion – after first choice of odor 2 (clove)	28.50 ± 4.76	22.82 ± 3.95	0.4786 Mann-Whitney
	**DA-Tsc1 KO/ Rptor WT**	**DA-Tsc1 KO/ Rptor Het**	**KO/WT vs KO/Het**
	Mean ± SEM	Mean ± SEM	adjusted p-value (Sidak's multiple

			comparisons test)
Perseverative errors	17.75 ± 4.07	10.27 ± 1.99	**0.0072**
Regressive errors	8.63 ± 2.16	6.55 ± 1.61	0.8944
Novel errors	1.63 ± 0.86	0.45± 0.21	0.9907
Irrelevant errors	2.88 ± 0.64	1.46 ± 0.58	0.9778
Omissions	0.63 ± 0.50	0.00 ± 0.00	0.9995

Supplementary Table 6. Summary of behavior testing results by sex, related to Supplementary Figure 4 (source data are provided as a Source Data file)

	**Male**	**Female**	**Male vs Female within genotype**
	Mean ± SEM	Mean ± SEM	p-value/ test
**Open Field Test** (males: n=15 WT and 20 KO mice, except rears n=22 KO; females: n=13 WT and 13 KO mice)			
Distance travelled (m) – DA-Tsc1 WT	93.14 ± 11.12	92.70 ± 8.88	0.9493 Mann-Whitney
Distance travelled (m) – DA-Tsc1 KO	93.21 ± 7.43	84.63 ± 10.38	0.1905 Mann-Whitney
Travelling speed (cm/s) – DA-Tsc1 WT	2.59 ± 0.31	2.57 ± 0.25	0.9909 Mann-Whitney
Travelling speed (cm/s) – DA-Tsc1 KO	2.59 ± 0.20	2.35 ± 0.29	0.1602 Mann-Whitney
Number of rears – DA-Tsc1 WT	538.10 ± 95.08	464.20 ± 51.42	0.5187 Unpaired t-test
Number of rears – DA-Tsc1 KO	446.80 ± 41.73	344.50 ± 61.31	0.0741 Mann-Whitney
Time spent in the center (s) – DA-Tsc1 WT	741.80 ± 89.45	520.40 ± 82.29	0.0876 Mann-Whitney
Time spent in the center (s) – DA-Tsc1 KO	469.00 ± 61.94	533.30 ± 92.01	0.5513 Unpaired t-test
Number of center entries – DA-Tsc1 WT	203.70 ± 22.85	163.3 ± 20.06	0.1269 Mann-Whitney
Number of center entries - DA-Tsc1 KO	170.80 ± 20.92	160.30 ± 25.94	0.6961 Mann-Whitney
**Open Field Test** (males: n=12 WT and 11 KO mice; females: n=4 WT and 6 KO mice)			
Time spent grooming (s) – DA-Tsc1 WT	139.70 ± 25.13	117.30 ± 19.40	0.6318 Unpaired t-test
Time spent grooming (s) – DA-Tsc1 KO	134.80 ± 29.75	104.40 ± 26.57	0.5116 Unpaired t-test
**Home Cage Observation** (males: n=3 WT and 10 KO mice; females: n=8 WT and 8 KO mice)			
Distance travelled (m) – DA-Tsc1 WT	106.80 ± 26.12	86.32 ± 17.32	0.4970 Mann-Whitney
Distance travelled (m) – DA-Tsc1 KO	91.27 ± 19.00	80.64 ± 15.10	0.9355 Mann-Whitney
Travelling speed (cm/s) – DA-Tsc1 WT	2.97 ± 0.73	2.40 ± 0.48	0.4061 Mann-Whitney
Travelling speed (cm/s) – DA-Tsc1 KO	2.53 ± 0.53	2.25 ± 0.42	0.9451 Mann-Whitney
**Home Cage Observation** (males: n=3 WT and 7 KO mice; females: n=9 WT and 7 KO mice)			
Number of rears – DA-Tsc1 WT	31.33 ± 8.84	51.33 ± 2.85	**0.0153** Unpaired t-test

Number of rears – DA-Tsc1 KO	29.71 ± 2.84	45.00 ± 8.29	0.1068<br>Unpaired t-test
Number of grooming bouts– DA-Tsc1 WT	8.33 ± 5.04	5.00 ± 1.25	0.3540<br>Unpaired t-test
Number of grooming bouts– DA-Tsc1 KO	10.43 ± 2.91	4.86 ± 1.64	0.1212<br>Unpaired t-test
Number of facial grooming bouts– DA-Tsc1 WT	4.67 ± 3.71	6.89 ± 0.95	0.4059<br>Unpaired t-test
Number of facial grooming bouts– DA-Tsc1 KO	7.43 ± 1.46	7.43 ± 1.48	0.9999<br>Unpaired t-test
Number of taffy pulling occurrences– DA-Tsc1 WT	3.33 ± 0.88	3.44 ± 1.16	0.9592<br>Unpaired t-test
Number of taffy pulling occurrences– DA-Tsc1 KO	1.86 ± 0.59	2.43 ± 0.95	0.6188<br>Unpaired t-test
Number of circling occurrences– DA-Tsc1 WT	3.00 ± 2.08	0.55 ± 0.24	0.0611<br>Unpaired t-test
Number of circling occurrences– DA-Tsc1 KO	1.14 ± 0.59	1.00 ± 0.38	0.8427<br>Unpaired t-test
Number of route tracing occurrences– DA-Tsc1 WT	2.00 ± 1.00	0.78 ± 0.32	0.1464<br>Unpaired t-test
Number of route tracing occurrences– DA-Tsc1 KO	0.00 ± 0.00	0.86 ± 0.55	0.1473<br>Unpaired t-test
Number of jumping occurrences– DA-Tsc1 WT	3.33 ± 3.33	15.22 ± 5.43	0.2557<br>Unpaired t-test
Number of jumping occurrences– DA-Tsc1 KO	2.71 ± 1.25	10.86 ± 9.13	0.3942<br>Unpaired t-test
Number of sniffing occurrences– DA-Tsc1 WT	48.67 ± 8.17	66.33 ± 3.72	**0.0488**<br>Unpaired t-test
Number of sniffing occurrences– DA-Tsc1 KO	42.14 ± 3.78	63.14 ± 6.68	**0.0181**<br>Unpaired t-test
Number of gnawing occurrences– DA-Tsc1 WT	1.67 ± 0.33	0.67 ± 0.47	**0.0455**<br>Mann-Whitney
Number of gnawing occurrences– DA-Tsc1 KO	1.00 ± 0.53	1.57 ± 0.78	0.5577<br>Unpaired t-test
**Elevated Plus Maze** (males: n=11 WT and 10 KO mice; females: n=4 WT and 6 KO mice)			
Closed arm entries – DA-Tsc1 WT	21.55 ± 2.93	27.00 ± 2.52	0.3110<br>Unpaired t-test
Closed arm entries – DA-Tsc1 KO	24.50 ± 2.27	19.67 ± 3.45	0.2420<br>Unpaired t-test
Open arm entries – DA-Tsc1 WT	15.09 ± 2.97	10.50 ± 2.06	0.3908<br>Unpaired t-test
Open arm entries – DA-Tsc1 KO	9.60 ± 1.72	11.00 ± 2.25	0.6276<br>Unpaired t-test
Closed arm time (s) – DA-Tsc1 WT	166.00 ± 16.69	189.20 ± 18.44	0.4563<br>Unpaired t-test
Closed arm time(s) – DA-Tsc1 KO	197.60 ± 16.68	169.60 ± 23.63	0.3380<br>Unpaired t-test
Open arm time (s) – DA-Tsc1 WT	39.45 ± 10.59	21.60 ± 6.89	0.3496<br>Unpaired t-test
Open arm time (s) – DA-Tsc1 KO	23.66 ± 9.57	60.48 ± 27.57	0.2630<br>Mann-Whitney

**Three Chamber Test** (males: n=5 Control and 5 KO mice; females: n=9 Control and 10 KO mice)			
Time in chamber (s) – Novel object Controls	216.50 ± 21.82	161.10 ± 26.53	0.1861 Unpaired t-test
Time in chamber (s) – Novel object DA-Tsc1 KO	163.70 ± 31.37	145.8 ± 33.87	0.7423 Unpaired t-test
Time sniffing (s) – Novel object Controls	17.85 ± 3.87	17.97 ± 2.91	0.9817 Unpaired t-test
Time sniffing (s) – Novel object DA-Tsc1 KO	14.02 ± 3.42	13.48 ± 2.70	0.9069 Unpaired t-test
Time in chamber (s) – Novel mouse Controls	314.70 ± 29.17	306.6 ± 51.14	0.9135 Unpaired t-test
Time in chamber (s) – Novel mouse DA-Tsc1 KO	316.20 ± 13.71	297.80 ± 42.59	0.7712 Unpaired t-test
Time sniffing (s) – Novel mouse Controls	64.93 ± 8.91	48.53 ± 8.63	0.2452 Unpaired t-test
Time sniffing (s) – Novel mouse DA-Tsc1 KO	57.32 ± 10.15	45.11 ± 7.75	0.3694 Unpaired t-test
	**Male**	**Female**	**Male vs Female**
	Mean ± SEM	Mean ± SEM	adjusted p-value (Sidak's multiple comparisons test)
**Rotarod Test** (males: n=8 WT and 7 KO mice; females: n=4 WT and 6 KO mice)			
Terminal speed trial 1 – DA-Tsc1 WT (rpm)	11.83 ± 1.96	9.25 ± 0.95	0.9999
Terminal speed trial 1 – DA-Tsc1 KO (rpm)	13.60 ± 2.98	14.00 ± 2.71	0.9999
Terminal speed trial 2 – DA-Tsc1 WT (rpm)	13.50 ± 2.60	14.75 ± 2.78	0.9999
Terminal speed trial 2 – DA-Tsc1 KO (rpm)	19.50 ± 5.75	13.33 ± 2.06	0.9899
Terminal speed trial 3 – DA-Tsc1 WT (rpm)	17.99 ± 5.72	17.25 ± 2.78	0.9999
Terminal speed trial 3 – DA-Tsc1 KO (rpm)	17.29 ± 1.86	15.67 ± 2.16	0.9999
Terminal speed trial 4 – DA-Tsc1 WT (rpm)	15.38 ± 2.47	21.25 ± 2.81	0.9726
Terminal speed trial 4 – DA-Tsc1 KO (rpm)	17.86 ± 2.12	21.20 ± 4.76	0.9999
Terminal speed trial 5 – DA-Tsc1 WT (rpm)	19.49 ± 3.31	20.50 ± 2.90	0.9999
Terminal speed trial 5 – DA-Tsc1 KO (rpm)	19.71 ± 2.89	21.60 ± 4.59	0.9999
Terminal speed trial 6 – DA-Tsc1 WT (rpm)	19.38 ± 3.21	21.50 ± 1.44	0.9999
Terminal speed trial 6 – DA-Tsc1 KO (rpm)	17.90 ± 3.35	21.67 ± 4.32	0.9996
Terminal speed trial 7 – DA-Tsc1 WT (rpm)	25.00 ± 4.02	24.00 ± 0.82	0.9999
Terminal speed trial 7 – DA-Tsc1 KO (rpm)	20.14 ± 2.88	26.50 ± 4.57	0.9583
Terminal speed trial 8 – DA-Tsc1 WT (rpm)	28.75 ± 3.47	28.25 ± 2.29	0.9999
Terminal speed trial 8 – DA-Tsc1 KO (rpm)	26.71 ± 4.36	26.33 ± 5.19	0.9999
Terminal speed trial 9 – DA-Tsc1 WT (rpm)	31.13 ± 2.75	28.25 ± 2.95	0.9999
Terminal speed trial 9 – DA-Tsc1 KO (rpm)	30.00 ± 3.59	27.50 ± 4.95	0.9999
Terminal speed trial 10 – DA-Tsc1 WT (rpm)	29.00 ± 4.04	23.57 ± 3.10	0.9679
Terminal speed trial 10 – DA-Tsc1 KO (rpm)	23.00 ± 1.47	26.67 ± 5.23	0.9999

Terminal speed trial 11 – DA-Tsc1 WT (rpm)	33.88 ± 3.52	27.00 ± 2.80	0.9167
Terminal speed trial 11 – DA-Tsc1 KO (rpm)	28.43 ± 4.20	26.33 ± 4.71	0.9999
Terminal speed trial 12 – DA-Tsc1 WT (rpm)	32.75 ± 4.33	32.75 ± 4.60	0.9999
Terminal speed trial 12 – DA-Tsc1 KO (rpm)	27.43 ± 3.37	26.50 ± 4.77	0.9999

Supplementary Table 7. Table of transgenic mouse lines

Mouse strain	Description	Reference
DAT^{IRES}*Cre*	Cre recombinase knocked in to the *Slc6a3* (DAT) locus	[1]
Tsc1$^{fl/fl}$	Conditional *Tsc1* knock-out with *loxP* sites flanking exons 17 and 18 of the *Tsc1* gene	[2]
Rptor$^{fl/fl}$	Conditional *Rptor* knock-out with loxP sites flanking exon 6 of the *Rptor* gene	[3]
Ai9	Cre reporter allele with CAG promoter lox-STOP-lox tdTomato cassette in the *Rosa26* locus	[4]

Supplementary Table 8. Primers used for mouse genotyping

Primer name	Sequence (5’ to 3’)
DAT^IRES^Cre WT rev	GGA CAG GGA CAT GGT TGA CT
DAT^IRES^Cre Mut rev	CCA AAA GAC GGC AAT ATG GT
DAT^IRES^Cre common for	TGG CTG TTG GTG TAA AGT GG
Tsc1 for	GTC ACG ACC GTA GGA GAA GC
Tsc1 rev	GAA TCA ACC CCA CAG AGC AT
Rptor for	AGCCTTTAGTACCCACTTGGC
Rptor rev	GGCATCTCACAAAGGGTACAG
Ai9 WT for	AAG GGA GCT GCA GTG GAG TA
Ai9 WT rev	CCG AAA ATC TGT GGG AAG TC
Ai9 Mut for	CTG TTC CTG TAC GGC ATG G
Ai9 Mut rev	GGC ATT AAA GCA GCG TAT CC

Supplementary Table 9. Antibodies used for immunohistochemistry

Antibody	Supplier and catalog #	Dilution
mouse anti-tyrosine hydroxylase	Immunostar: 22941	1:1000
rabbit anti-phospho-S6 (Ser240/244)	Cell Signaling: 5364S	1:1000
Alexa-488 goat anti-mouse	Thermo Fisher: A-11001	1:500
Alexa-633 goat anti-rabbit	Thermo Fisher: A-11034	1:500

Supplementary Table 10. Antibodies used for western blotting

Antibody	Supplier and catalog #	Dilution
mouse anti-tyrosine hydroxylase	Immunostar: 22941	1:3000
mouse anti-DARPP-32	gift from Dr. Paul Greengard's Lab[5]	1:3000
mouse anti-Histone-3	Cell Signaling: 96C10	1:1500
rabbit anti-VMAT2	Alomone Labs: AMT-006	1:1400
goat anti-rabbit HRP	Bio-Rad: 170-5046	1:5000
goat anti-mouse HRP	Bio-Rad: 170-5047	1:5000

Supplementary References

1. Backman CM*, et al.* Characterization of a mouse strain expressing Cre recombinase from the 3' untranslated region of the dopamine transporter locus. *Genesis* **44**, 383-390 (2006).

2. Kwiatkowski DJ*, et al.* A mouse model of TSC1 reveals sex-dependent lethality from liver hemangiomas, and up-regulation of p70S6 kinase activity in Tsc1 null cells. *Hum Mol Genet* **11**, 525-534 (2002).

3. Sengupta S, Peterson TR, Laplante M, Oh S, Sabatini DM. mTORC1 controls fasting-induced ketogenesis and its modulation by ageing. *Nature* **468**, 1100-1104 (2010).

4. Madisen L*, et al.* A robust and high-throughput Cre reporting and characterization system for the whole mouse brain. *Nature neuroscience* **13**, 133-140 (2010).

5. Hemmings HC, Jr., Greengard P. DARPP-32, a dopamine- and adenosine 3':5'-monophosphate-regulated phosphoprotein: regional, tissue, and phylogenetic distribution. *J Neurosci* **6**, 1469-1481 (1986).
