# Supplementary material for: Tsc1-mTORC1 signaling controls striatal dopamine release and cognitive flexibility
Source: Nat Commun. 2019 Nov 28;10:5426. doi: 10.1038/s41467-019-13396-8 (PMC6882901; doi:10.1038/s41467-019-13396-8)
Supplement: Supplementary file 2 — Reporting Summary [file 41467_2019_13396_MOESM2_ESM.pdf]

nature research

Corresponding author(s): Helen Bateup

Last updated by author(s): Oct 25, 2019

Reporting Summary

Nature Research wishes to improve the reproducibility of the work that we publish. This form provides structure for consistency and transparency in reporting. For further information on Nature Research policies, see Authors & Referees and the Editorial Policy Checklist.

Statistics

For all statistical analyses, confirm that the following items are present in the figure legend, table legend, main text, or Methods section.

n/a	Confirmed	
☐	☒	The exact sample size (*n*) for each experimental group/condition, given as a discrete number and unit of measurement
☐	☒	A statement on whether measurements were taken from distinct samples or whether the same sample was measured repeatedly
☐	☒	The statistical test(s) used AND whether they are one- or two-sided<br>*Only common tests should be described solely by name; describe more complex techniques in the Methods section.*
☒	☐	A description of all covariates tested
☐	☒	A description of any assumptions or corrections, such as tests of normality and adjustment for multiple comparisons
☐	☒	A full description of the statistical parameters including central tendency (e.g. means) or other basic estimates (e.g. regression coefficient) AND variation (e.g. standard deviation) or associated estimates of uncertainty (e.g. confidence intervals)
☒	☐	For null hypothesis testing, the test statistic (e.g. *F*, *t*, *r*) with confidence intervals, effect sizes, degrees of freedom and *P* value noted<br>*Give P values as exact values whenever suitable.*
☒	☐	For Bayesian analysis, information on the choice of priors and Markov chain Monte Carlo settings
☒	☐	For hierarchical and complex designs, identification of the appropriate level for tests and full reporting of outcomes
☒	☐	Estimates of effect sizes (e.g. Cohen's *d*, Pearson's *r*), indicating how they were calculated

Our web collection on statistics for biologists contains articles on many of the points above.

Software and code

Policy information about availability of computer code

Data collection	Confocal microscopy images were acquired using Zen Black (Zeiss, release version 14). Physiology data were acquired using ScanImage software (https://github.com/bernardosabatini/SabalabAcq) in Matlab R2009b (MathWorks). Voltammetry data was collected using AxoScope 10.5 (Molecular Devices). HPLC data was collected with Clarity (DataApex, release version 2.6). Electron micrographs were acquired using Digital Micrograph (GATAN, release version 3) software for dorsal striatum and Radius (EMSIS, release version 2) software for ventral striatum. Behavior data for the open field, 3-chamber test, and elevated plus maze were acquired using AnyMaze (Stoelting, version 4.99). Rotarod data was acquired with default software for Ugo Basile 47650 rotarod apparatus. Details on data collection for each experiment are provided in the Methods.
Data analysis	Confocal microscopy images were analyzed in Image J (NIH, version 1.5). Sholl analysis was performed using IMARIS software (Bitplane, release versions 8 and 9). Physiology data were analyzed in Igor Pro versions 6 and 8 (Wavemetrics). Voltammetry data was analyzed in AxoScope 10.6 (Molecular Devices) and Microsoft Excel (2013). Western blots were analyzed using Image J (NIH, Version 1.5). HPLC data was analyzed in Microsoft Excel (2013). Electron micrographs were analyzed using ImageJ (NIH, version 1.5) and software designed by Dr. Max Larsson, available at: https://old.liu.se/medfak/forskning/larsson-max/software?l=en. Additional analysis was performed in Microsoft Excel (2016). Behavior data for the open field, 3-chamber test, and elevated plus maze were analyzed using AnyMaze (Stoelting, version 4.99). Other behavior data was analyzed in Microsoft Excel (2013). Graphing and statistical analysis was done in GraphPad Prism versions 6 and 8. Details on quantification and data analysis for each experiment are provided in the Methods.

For manuscripts utilizing custom algorithms or software that are central to the research but not yet described in published literature, software must be made available to editors/reviewers. We strongly encourage code deposition in a community repository (e.g. GitHub). See the Nature Research guidelines for submitting code & software for further information.

Data

Policy information about availability of data

All manuscripts must include a data availability statement. This statement should provide the following information, where applicable:

- Accession codes, unique identifiers, or web links for publicly available datasets
- A list of figures that have associated raw data
- A description of any restrictions on data availability

Source data underlying all figures and tables has been provided in the Source Data file.

Field-specific reporting

Please select the one below that is the best fit for your research. If you are not sure, read the appropriate sections before making your selection.

☒ Life sciences ☐ Behavioural & social sciences ☐ Ecological, evolutionary & environmental sciences

For a reference copy of the document with all sections, see nature.com/documents/nr-reporting-summary-flat.pdf

Life sciences study design

All studies must disclose on these points even when the disclosure is negative.

Sample size	No sample-size calculations were performed. Sample size was determined to be adequate based on prior literature and the magnitude and consistency of measurable differences between groups.
Data exclusions	For physiology data, cells were excluded if they failed quality control checks (e.g. series resistance > 30 mOhms). No other data were excluded.
Replication	No replication attempts were made.
Randomization	For the rapamycin experiments, mice were randomly chosen to receive vehicle or rapamycin. For all other experiments, randomization was not applicable as the groups were determined by genotype.
Blinding	Experimenters were blinded to animals' genotypes for histology, electron microscopy, HPLC, western blotting, and behavioral experiments. Rapamycin histology experiments were performed blind to genotype and treatment. All Tsc1;Raptor;DAT-Cre voltammetry experiments were performed blind to genotype. Other experiments were not performed blind.

Reporting for specific materials, systems and methods

We require information from authors about some types of materials, experimental systems and methods used in many studies. Here, indicate whether each material, system or method listed is relevant to your study. If you are not sure if a list item applies to your research, read the appropriate section before selecting a response.

Materials & experimental systems

n/a	Involved in the study	
☐	☒	Antibodies
☒	☐	Eukaryotic cell lines
☒	☐	Palaeontology
☐	☒	Animals and other organisms
☒	☐	Human research participants
☒	☐	Clinical data

Methods

n/a	Involved in the study	
☒	☐	ChIP-seq
☒	☐	Flow cytometry
☒	☐	MRI-based neuroimaging

Antibodies

Antibodies used

Tyrosine hydroxylase, Immunostar, #22941 (for IHC and western)
Tyrosine hydroxylase, Chemicon, #AB152 (for EM analysis)
Goat-anti-rabbit gold-conjugated, Nanoprobes,#2003 (1.4 nm Nanogold) (For EM analysis)
Phospho-S6 (ser240/244), Cell Signaling, #5364
DARPP-32, gift from Dr. Paul Greengard's lab
Histone 3, Cell Signaling, #96C10
VMAT2, Alomone Labs, #AMT-006
Goat anti-rabbit HRP, Bio-Rad, #170-5046
Goat anti-mouse HRP, Bio-Rad, 170-5047
Goat anti-mouse Alexa 488, ThermoFisher, #A11001

	Goat anti-rabbit Alexa 633, ThermoFisher, #A11034<br>Goat anti-mouse Alexa 633, ThermoFisher, #A21050
Validation	Commercial antibodies were validated by the manufacturer. The custom generated DARPP-32 antibody was validated using tissue from DARPP-32 KO mice and as reported in Hemmings & Greengard, J Neurosci, 1986.

Animals and other organisms

Policy information about studies involving animals; ARRIVE guidelines recommended for reporting animal research

Laboratory animals	Laboratory mice of the following strains were used: Tsc1fl/fl;DAT-IRES-Cre (with and without the Ai9 Cre-reporter strain) and Tsc1fl/fl;Raptorfl/fl;DAT-IRES-Cre (with and without the Ai9 Cre-reporter strain). Both males and females were used. Mice were 8-12 weeks old, except for the voltammetry experiments with aged mice, which were 21-24 months old.
Wild animals	The study did not involve wild animals.
Field-collected samples	The study did not involve samples collected from the field.
Ethics oversight	All animal procedures were carried out in accordance with protocols approved by the University of California, Berkeley Institutional Animal Care and Use Committee (IACUC).

Note that full information on the approval of the study protocol must also be provided in the manuscript.
